# Gal-3 Deficiency Suppresses *Novosphyngobium aromaticivorans* Inflammasome Activation and IL-17 Driven Autoimmune Cholangitis in Mice

**DOI:** 10.3389/fimmu.2019.01309

**Published:** 2019-06-07

**Authors:** Aleksandar Arsenijevic, Jelena Milovanovic, Bojana Stojanovic, Dragana Djordjevic, Ivan Stanojevic, Nenad Jankovic, Danilo Vojvodic, Nebojsa Arsenijevic, Miodrag L. Lukic, Marija Milovanovic

**Affiliations:** ^1^Center for Molecular Medicine and Stem Cell Research, Faculty of Medical Sciences, University of Kragujevac, Kragujevac, Serbia; ^2^Faculty of Medical Sciences, Institute of Histology, University of Kragujevac, Kragujevac, Serbia; ^3^Faculty of Medical Sciences, Institute of Pathophysiology, University of Kragujevac, Kragujevac, Serbia; ^4^Department of Pharmacy, Faculty of Medical Sciences, University of Kragujevac, Kragujevac, Serbia; ^5^Institute of Medical Research, Faculty of Medicine, Military Medical Academy, Belgrade, Serbia; ^6^Department of Chemistry, Faculty of Science, University of Kragujevac, Kragujevac, Serbia

**Keywords:** primary biliary cholangitis, galectin-3, *Novosphingobium aromaticivorans*, C57BL/6 mice, NLRP3, galectin-3 inhibitor

## Abstract

Gal-3 has the role in multiple inflammatory pathways. Multiple-hit etiology of primary biliary cholangitis (PBC) and evolving immune response at various stages of the disease includes involvement of Gal-3 in PBC pathogenesis. In this study we aimed to clarify the role of Gal-3 in *Novosphingobium aromaticivorans* (*N. aromaticivorans*) induced biliary disease. Autoimmune cholangitis was induced in mice by two intra-peritoneal injections of *N. aromaticivorans* within 2 weeks. The role of Gal-3 was evaluated by using Lgals3^−/−^ mice and mice treated with Gal-3 inhibitor. The histological and serological parameters of disease, phenotype of dendritic, NK, NKT, and T cells and inflammasome expression were evaluated. Marked attenuation of the disease in Lgals3^−/−^ and Gal-3 inhibitor, DAVANAT^®^, treated mice is manifested by the absence of bile duct damage, granulomas and fibrosis. Liver infiltrates of *N. aromaticivorans* infected wild type mice had higher incidence of pro-inflammatory macrophages, dendritic cells, NK, NKT, and T cells. Lgals3 deletion and treatment with Gal-3 inhibitor reduced inflammatory mononuclear cell infiltrate, expression of NLRP3 inflammasome in the liver infiltrates and interleukin-1β (IL-1β) production in the livers of *N. aromaticivorans* infected mice. *In vitro* stimulation of wild type peritoneal macrophages with *N. aromaticivorans* caused increased NLRP3 expression, caspase-1 activity and IL-1β production compared with Lgals3^−/−^ cells. Our data highlight the importance of Gal-3 in promotion of inflammation in *N. aromaticivorans* induced PBC by enhancing the activation of NLRP3 inflammasome and production of IL-1β and indicate Gal-3 as possible therapeutical target in autoimmune cholangitis. Galectin-3 appears involved in inflammatory response to gut commensal leading to PBC.

## Introduction

Primary biliary cholangitis is an organ-specific autoimmune disease, mediated by immune mechanisms that lead to selective destruction of intrahepatic bile ducts. Although a multi-lineage response, including involvement of autoantibodies and CD4^+^ and CD8^+^ T cells, against the immunodominant autoantigen PDC-E2 (1, 2) is central component of disease pathogenesis (3), different components of innate immunity play important roles in different phases of the disease (4, 5).

Gal-3 emerged as a multifunctional glycoprotein with diverse functions, immunomodulatory, role in control of cell death, activation, differentiation and migration, all critical for immune and inflammatory responses (6). Further, Gal-3 as other molecules involved in cellular homeostasis, under altered conditions, such as altered cytokine microenvironment, can become hazardous. Gal-3 may have double role in autoimmune processes depending on the dominant pathogenic mechanisms involved (7). Further, depending on the stage of disease which is most affected, Gal-3 can have the opposite effects on the outcome of the same disease. Gal-3 inhibits apoptosis and thus reduces the autoantigen release and attenuates systemic lupus erythematous (SLE), but on the other hand Gal-3 can enhance type I IFN responses, thereby worsening autoimmune reactions in SLE (8).

Our initial research on the role of Gal-3 in PBC pathogenesis has demonstrated that Gal-3 deletion exacerbates cholangitis in 2-octynoic acid coupled to BSA immunized C57BL/6 mice by enhancing apoptosis of cholangiocytes followed by enhanced autoantigen release and increased stimulation of antigen presenting cells (9). Recent report indicates pro-inflammatory role of Gal-3 in dnTGF-βRII mice that spontaneously develop autoimmune cholangitis (10). Therefore, it was of interest to evaluate the role of Gal-3 in another model of PBC that can be induced in mice by infection with ubiquitous, aerobic, free-living Gram-negative bacterium *N. aromaticivorans* (11). Since *N. aromaticivorans* is commensal of digestive tract mucosa, autoimmune cholangitis developed in mice after infection with *N. aromaticivorans* is the most similar to pathogenetic mechanisms of PBC in humans.

*N. aromaticivorans* contains glycosphingolipids in cell wall instead of LPS (12) which presented in complex with CD1d molecules on dendritic cells activate CD1d-restricted NKT cells (13). The central role in activation of autoreactive cells in xenobiotic induced PBC have myeloid derived cytokines (14) while in *N. aromaticivorans* induced model indispensable role in induction of autoimmune process NKT cells play (11).

In order to further explore the role of Gal-3 in development and progression of the autoimmune cholangitis and to envisage the potential novel therapeutic strategies, we used Gal-3 deficient mice and Gal-3 inhibitor treatment in *N. aromaticivorans* induced PBC. We report herein that Gal-3 deletion and Gal-3 inhibitor treatment prevents bile duct damage in bacteria induced PBC. Our findings indicate that Gal-3 deficiency results in reduced inflammasome activation with *N. aromaticivorans*, and subsequent blockade of autoimmune process.

## Materials and Methods

### Animals

Female Lgals3^−/−^ mice on the C57BL/6 background and wild-type (WT) C57BL/6 mice, 8 weeks of age, were used for the experiments. Knockout mice were obtained from the University of California, Davis (Davis, CA; by courtesy of D.K. Hsu and F.T. Liu). For galectin-3 genotyping two individual primers, with a common downstream primer of 5-CACTCTCAAAGGGGAAGGCTGACTGTC3; wild-type allele, 5-TAGGTGAGAGTCACAAGCTGGAGGCC-3, which produced a 490-bp fragment; mutant allele, 5-GGCTGACCGCTTCCTCGTGCTTTACGG3, which amplified a 300-bp segment from the neomycin gene were used. All mice were housed in a temperature–controlled environment with a 12-h light–dark cycle and were administered standard laboratory chow and water *ad libitum*. Animals were matched by age and weight within the each experiment. All of the animal procedures were approved by the Ethics Committee of Faculty of Medical Sciences, University of Kragujevac.

### Induction of PBC Like Disease

*N. aromaticivorans* (ATCC 700278), was grown overnight in Trypticase Soy broth, diluted in fresh medium, grown for 8 h at 37°C, washed, diluted in PBS and cell density was determined by dark filed microscopy using Neubauer counting chamber. Bacterial suspension (100 μl) containing 5 × 10^7^ *Novosphyngobium aromaticivorans* CFU was injected intravenously on day 0 and on day 14.

### Anti-PDC-E2 ELISA

Blood samples were collected from the facial vein at weeks 2, 4, and 8 after intravenous application of *N. aromaticivorans*, and tested for levels of anti-PDC-E2 antibodies using an enzyme-linked immunosorbent assay (ELISA) as previously described (9). Briefly, 96-well ELISA plates were coated with 10 μg/ml of purified recombinant PDC-E2 in 100 μl of carbonate buffer (pH 9.6) at 4°C overnight, washed with Tris-Buffered Saline Tween-20 (TBS-T), and blocked with 5% skim milk in TBS for 30 min. One hundred microliters of diluted sera (1:250) were added to each well and incubated for 2 h at room temperature. After washing, horseradish peroxidase (HRP)-conjugated anti-mouse immunoglobulin (A + M + G) (H + L) (1:3,000) (INVITROGEN ZyMax™) was added. The plates were incubated for 1 h at room temperature, the plates were re-washed and color developed with 100 μl of TMB peroxidase substrate (BD Biosciences, San Jose, CA) added to each well. Optical density (OD) was read at 450 nm at Zenyth multimode detector 3100. Previously calibrated positive and negative standards were included with each assay.

### Biochemistry

Alanine transaminase (ALT) and aspartate transaminase (AST) values in serum were determined by (AST or SGOT) Activity Colorimetric Assay Kit and Alanine Aminotransferase (ALT or SGPT) Activity Colorimetric/Fluorometric Assay Kit (ELAB Science) according to the manufacturer's instructions. Optical density (OD) was read at 450 nm at Zenyth multimode detector 3100 and serum levels were expressed as mU/ml.

### Liver Histology Scoring

Liver tissues were fixed in 4% paraformaldehyde at room temperature for 2 days and embedded in paraffin. Paraffin blocks were brought to room temperature and sectioned on a rotary microtome (Leica RM2135). Twenty four serial 4-μm sections were floated onto water at 40°C before being transferred to glass microscope slides. The sections were deparaffinized, stained with hematoxylin and eosin (H&E) and every fourth (six slides) was evaluated for periportal inflammation, infiltration of bile ducts without damage, infiltration, and damage of bile ducts, and subcapsular infiltrates. Based on the levels of pathology, the indices were scored as 0, none; 1, mild; 2, moderate; 3, severe; and 4, very severe pathology. Histological score I was calculated as mean value of each scored index. Granulomas, and fibrosis were scored as 0, none; 1, mild; 2, moderate; and 3, severe pathology and histological score II was calculated based on these values. Histological analysis and scoring were performed in blinded fashion. Picrosirius red (Direct Red 80, Sigma-Aldrich, St. Louis, MO, USA) staining was used to assess hepatic collagen deposition. Quantification of fibrosis in mouse liver sections stained with picrosirius red (10 × ) was performed using ImageJ software (National Institutes of Health, Bethesda, MD, USA), on 10 fields/section. The images were captured with a light microscope (BX51; Olympus) equipped with a digital camera.

### Galectin-3 Inhibitor Injections

C57BL/6 WT mice were receiving intra-peritoneal injections of Gal-3 inhibitor, DAVANAT^®^, 300 μg per mouse (kindly provided by Professor Klyosov and Professor Traber from Galectin Therapeutics Inc., Newton, MA) three times weekly for 4 weeks, starting from the first dose of bacteria.

### Isolation of Hepatic Mononuclear Cells and Splenocytes and Flow Cytometry

The isolation of liver-infiltrating mononuclear cells was conducted as previously described (9). Spleens were excised and single-cell suspensions were obtained by mechanical disruption through 40-mm cell-strainer nylon mesh and lysis of erythrocytes. For cytofluorometry following antibodies were used CD3, CD4, CD8α, TCRβ, CD49b, NKG2D, perforin, CD1d, I-A/I-E, CD86, T-bet, GATA3, Foxp3, RoRγt, IL-1β, IL-6, IL-12, IL-17, IFN-γ, TNF-α, IL-4, IL-5, and IL-13 with conjugated fluorochromes (BD Biosciences), CD11c, CD11b, F4/80 (BioLegend, San Diego, CA), NLRP3 (R&D systems). Antibodies were incubated with cells for 30 min at 4°C, cells were washed twice in PBS and then analyzed. For detection of intracellular staining cells were fixed and permeabilized after cell-surface marker staining using Cytofix/Cytoperm Kit (BD Biosciences). For flow cytometric analysis of cytokines, cells were stimulated with Phorbol 12-myristate13-acetate (50 ng/ml) (Sigma, USA), Ionomycin (500 ng/ml) (Sigma, USA), and GolgyStop (BD Pharmingen) for 4 h. Isotype Abs with matching conjugates were used as negative controls, while unstained controls were used to determine expression of intracellular cytokines. Cells were analyzed with the FACSCalibur Flow Cytometer (BD Biosciences), and analysis was conducted with FlowJo software (Tree Star).

### *In vitro* Stimulation of Dendritic Cells and NK Cells With *N. aromaticivorans*

NK cells were isolated from livers of untreated WT and Lgals3^−/−^ mice by magnetic cell sorting, using Dynal® Mouse CD49b isolating kit (Invitrogen). Isolated NK cells were placed in 24-well plates (10,000 cells/well) and 100,000 *N. aromaticivorans* grown in Trypticase Soy broth were added. Dendritic cells were isolated from spleens of untreated WT and Lgals3^−/−^ mice using Dynabeads® Mouse DC Enrichment Kit (Invitrogen) and placed in 24-well plate (100.000 cells/well). *N. aromaticivorans* (1,000,000) were added. Dendritic and NK cells with bacteria were cultured in antibiotic-free complete DMEM. After a 24-h incubation at 37°C, dendritic and NK cells were washed in PBS and analyzed for expression of KLRG1, NKG2D, IFN-γ, IL-17 (NK cells) and CD86, IL-4, IL-12, NLRP3 (dendritic cells) by flow cytometry.

### Immunohistochemistry

Cryostat liver tissue sections (4 μm) were fixed and permeabilised in ice cold acetone. After washing and blocking with 2% bovine serum albumin the sections were incubated with primary mouse anti-Gal-3, primary rabbit anti-NLRP3 and primary rabbit anti-IL-1β (Abcam, Cambridge, UK) antibody. Staining was visualized by using rabbit specific HRP/AEC detection IHC Kit (Abcam, Cambridge, UK) for NLRP3 and IL-1β and EXPOSE mouse and rabbit specific HRP/DAB detection IHC Kit (Abcam, Cambridge, UK). Sections were photomicrographed with a digital camera mounted on light microscope (Olympus BX51, Japan) and analyzed (15). Analysis was performed on 10 fields/section (×40). Results are presented as percent of positive staining cells per infiltrate.

### Cytokine Measurements

The liver tissues were weighed and a 100 mg portion of the liver was homogenized in 0.5 mL PBS. Liver homogenates were centrifuged at 14,000 g for 10 min at 4°C. Supernatants were transferred to clean microcentrifuge tubes and stored at −20°C. Cytokine levels in liver supernatants were determined using mouse Duoset enzyme-linked immunosorbent assay (ELISA) kits for IL-1β (R&D Systems) according to the manufacturer's instructions.

### Stimulation of Peritoneal Macrophages With *N. aromaticivorans in vitro*

Macrophages were harvested from the peritoneal cavity of WT and Lgals3^−/−^ untreated mice using peritoneal lavage. Complete Dulbecco's modified Eagle's medium (6 ml) supplemented with FBS (10%), glutamine (2 mM), 100 U/ml penicillin, and 100 mg/ml streptomycin was injected into the peritoneal cavity and the medium containing cells was retrieved. The extracted cells were depleted of red blood cells using red blood cell lysis buffer containing 8.3 g/l ammonium chloride in 10 mM Tris-HCl, pH 7.5 and washed by centrifugation.

Cells were stimulated with *N. aromaticivorans* for 24 h (cell/bacteria ratio 1:10) at 37°C in a 5% CO_2_ incubator. Where indicated, cells were preincubated with the caspase-1 inhibitor Z-YVAD-FMK (10 μmol/L; Bachem AG, Bubendorf, Switzerland). After incubation, the cell supernatants were collected and cells were labeled with anti-F4/80 (BioLegend), anti-IL-1β (BD Pharmingen), and anti-NLRP3 (R&D systems) fluorochrome-conjugated monoclonal antibodies or isotype matched controls for flow cytometry. The levels of IL-1β and IL-6 in cell supernatants were determined using mouse Duoset ELISA kits (R&D Systems).

### Caspase-1 Activity Assay

Peritoneal macrophages were seeded on six-well plates (1 × 10^6^ cells/well), incubated with *N. aromaticivorans* (1 × 10^6^ cells/well) for 24 h, Caspase-1 activity in cell lysates was determined using the Caspase-1 Colorimetric Kit (R&D Systems) according to the manufacturer's recommendations.

### Isolation and *in vitro* Stimulation of Splenocytes

Spleens were removed from untretaed mice, minced in RPMI 1640 (Sigma Aldrich) and forced gently through 40-mm cell-strainer nylon mesh (Falcon) using a sterile syringe plunger and centrifuged at 300 g for 5 min. Pelleted cells were incubated with 2 ml ammonium chloride/Tris-chloride (pH 7.2) (erythrocyte lysing buffer) at room temperature for 5 min, then supplemented with 1 ml FBS, centrifuged subsequently at 300 g 5 min and resuspended in RPMI 1640 with 10% (vol/vol) FBS. Isolated splenocytes were plated at a density of 2.5 × 10^6^ cells/well, incubated with DAVANAT^®^ (100 μM) for 2 h and subsequently primed with 1 μg/ml Lipopolysaccharides, LPS (from E. coli 055:B5; L2880, Sigma-Aldrich) for 24 h, as described (16). The level of Gal-3 in cell culture supernatants was determined by Duoset ELISA kits (R&D Systems).

### Immunofluorescent Staining

For immunofluorescent microscopy the peritoneal cells harvested and resuspended in the above culture medium were seeded into 6-well plates (10^6^/well) containing coverslips. *N. aromaticivorans* (10^7^/well) were added and cells were cultured for 7 days. Immunofluorescent staining of peritoneal macrophages was performed using rabbit anti-mouse F4/80 (1:200) and mouse anti-mouse IL-1β (1:200) and NLRP3 (1:200) antibodies (Abcam), followed by incubation with PE conjugated anti-rabbit and FITC-conjugated anti-mouse IgG antibody (1:400; Abcam). The sections were mounted with ProLong Gold antifade reagent with DAPI (Invitrogen) and analyzed at ×200 magnification using the Olympus BX51 fluorescence microscope.

### RNA Extraction and Real-Time qRT-PCR

Total RNA was extracted from the frozen mouse livers using TRIzol (Invitrogen, Carlsbad, CA) according to the manufacturer's instructions. Total RNA (2 μg) was reverse-transcribed to cDNA using RevertAid H Minus First Strand cDNA Synthesis Kit (Thermo Fisher Scientific). qRT-PCR was performed using Luminaris Color HiGreen qPCR Master Mix (Thermo Fisher Scientific) and miRNA specific primers for NLRP3 (forward: GCCCTTGCCTGGAGGAGTCATG, reverse: CATTGAAGCGGGGGTTAAAGTGG), ASC (forward: GAGCAGCTGCAAACGACTAA, reverse: GTCCACAAAGTGTCCTGTTCTG) Procolagen α1 (forward: GCTCCTCTTAGGGGCCACT, reverse: CCACGTCTCACCATTGGGG), α-SMA (forward: ACTGGGACGACATGGAAAAG, reverse: CATCTCCAGAGTCCAGCACA), β-actin as a housekeeping gene (forward: AGCTGCGTTTTACACCCTTT, reverse: AAGCCATGCCAATGTTGTCT). qPCR reactions were initiated with a 10 min incubation time at 95°C followed by 40 cycles of 95°C for 15 s and 60°C for 60 s in a Mastercycler ep realplex (Eppendorf, Hamburg, Germany). Relative expression of genes was calculated according to the formula 2^−(Ct−Ctactin)^, where C_t_ is the cycle threshold of the gene of interest and C_tactin_ is the cycle threshold value of the housekeeping gene (ß-actin).

### Statistical Analysis

The data are presented as mean ± SD or mean ± SEM. Statistical significance was determined by Independent sample Student *t*-test and ANOVA, and, where appropriate, Mann-Whitney *U*-test or Kruskal-Wallis. Statistical significance was assumed at *p* < 0.05. Statistical analyses were performed using SPSS 13.0.

## Results

### Lgals3 Deletion Attenuates PBC Induced With *N. aromaticivorans*

**Figure 1:**
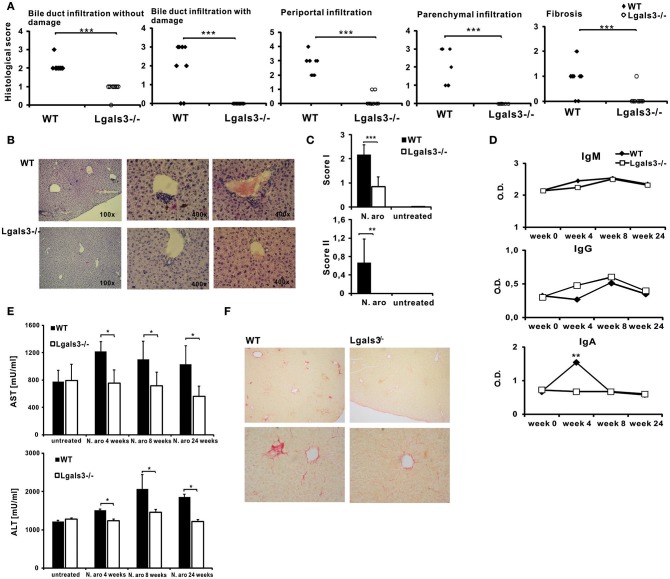
Deletion of Gal-3 significantly attenuates PBC in C57BL/6 mice. C57BL/6 WT and Lgals3^−/−^ mice received two intra-peritoneal injections of *N. aromaticivorans* (10^7^ CFU) and 8 weeks after liver tissues were fixed, sectioned and stained with hematoxylin and eosin **(A)** Histological score for parameters of liver inflammation, granuloma formation, and fibrosis presented per mouse. **(B)** Representative liver sections of immunized WT and Lgals3^−/−^ infected mice. **(C)** Total score I (liver infiltration and bile duct damage) and score II (liver fibrosis and granuloma formation). **(D)** Serum levels of anti-PDC-E2 antibodies in non-infected mice (week 0) and 4, 6, and 8 weeks after *N. aromaticivorans* infection. **(E)** Significant increase of levels of AST and ALT in sera of *N. aromaticivorans* infected WT mice. **(F)** Representative images of Picrosirius staining of liver sections obtained from WT and Lgals3^−/−^ mice 8 weeks after *N. aromaticivorans* infection. Data are from representative experiment, presented as mean + SE, 8 mice/group, *p < 0.05, **p < 0.005, ***p < 0.001.

All histological parameters indicating autoimmune cholangitis were seen in WT mice, 8 weeks after infection with *N. aromaticivorans* (Figure 1A). Lgals3^−/−^ mice didn't develop histological parameters of PBC (Figure 1). Bile duct damage was not detected in the livers of Lgals3^−/−^ mice (0/8) (Figure 1A). Weak periportal inflammation without parenchymal infiltration was detected in 2/8 Lgals3^−/−^ mice in contrast to 8/8 WT mice (Figure 1A). Fibrosis was detected in 1/8 Lgals3^−/−^ mice in contrast to 6/8 in WT mice (Figures 1A,F). Moderate to prominent periportal inflammation and dense periductal lymphoid infiltration with bile duct loss were detected in the livers of WT mice (Figure 1B). Only mild bile duct infiltration with single mononuclear cells around continuous bile duct epithelium was noticed in the livers of Lgals3^−/−^ mice (Figure 1B). Granulomas and subcapsular infiltration were not observed in any group of mice. In summary significantly higher score I (periportal inflammation, infiltration of bile ducts without damage, infiltration, and damage of bile ducts, and subcapsular infiltrates) (*p* < 0.001) and score II (granuloma formation and fibrosis) (*p* < 0.005) were noticed in the group of WT mice infected with *N. aromaticivorans* compared with infected Lgals3^−/−^ mice (Figure 1C). There was no significant difference in IgG and IgM levels between WT and Lgals3^−/−^ mice at any time of examination (Figure 1D). Four weeks after infection level of anti-PDC-E2 IgA in the serum of WT mice was significantly higher compared to the group of Lgals3^−/−^ mice (Figure 1D).

Levels of AST and ALT in the serum of infected WT mice were significantly higher 4 and 8 weeks after infection, in comparison with group of untreated mice (Figure 1E). Interestingly, no increase of AST and ALT after infection was observed in serum of Lgals3^−/−^ mice. Serum levels of AST and ALT after infection with *N. aromaticivorans* were significantly lower in the group of Lgals3^−/−^ mice in comparison with the group of WT mice (Figure 1E).

### Gal-3 Deficiency Significantly Attenuates Type 17 Immune Responses in Livers of *N. aromaticivorans* Infected Mice

**Figure 2:**
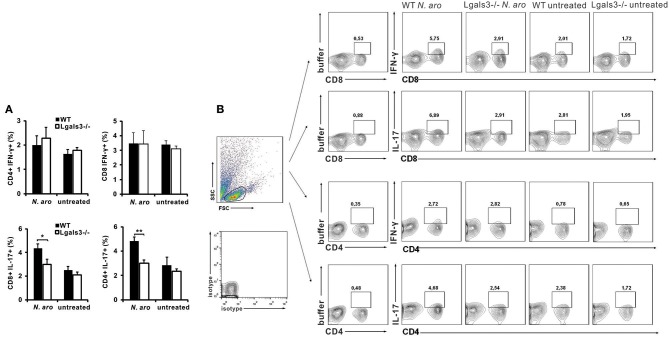
Gal-3 deletion decreases Th17 and Tc17 lymphocytes in livers after PBC induction. Eight weeks after *N. aromaticivorans* infection mononuclear cells were isolated from livers and used for flow cytometric analysis after intracellular staining for inflammatory cytokines. **(A)** Percentages of CD4+ and CD8+ cells containing IL-17 and IFN-γ in the liver in WT and Lgals3^−/−^ mice. **(B)** Representative dot plots of CD4+ and CD8+ cells positive for IFN-γ and IL-17. Data are presented as mean+SE, 8 mice/group, *p < 0.05, **p < 0.005.

In order to evaluate eventual impact of Gal-3 on the composition of lymphocytic infiltrates in the livers, flow cytometric analysis of mononuclear cells isolated from the livers was done. Eight weeks after *N. aromaticivorans* infection significantly lower percentage of IL-17 expressing CD4+ cells (*p* < 0.005) and IL-17 expressing CD8 cells (*p* < 0.05) was found in group of infected Lgals3^−/−^ mice compared with group of infected WT mice (Figures 2A,B). There was no significant difference in the percentage of CD4+ and CD8+ cells containing IFN-γ in the livers of infected Lgals3^−/−^ and WT mice and no increase in comparison with control animals. No significant difference in percentage of both IL-17 and IFN-γ positive cells in CD4+ and CD8+ populations between infected WT and Lagls3^−/−^ mice was found (Supplementary Figure 1).

### Gal-3 Deficiency Significantly Reduces Liver Infiltration With Activated, Cytotoxic, and Inflammatory NK Cells and Affects Their Inflammatory and Cytotoxic Capacity

**Figure 3:**
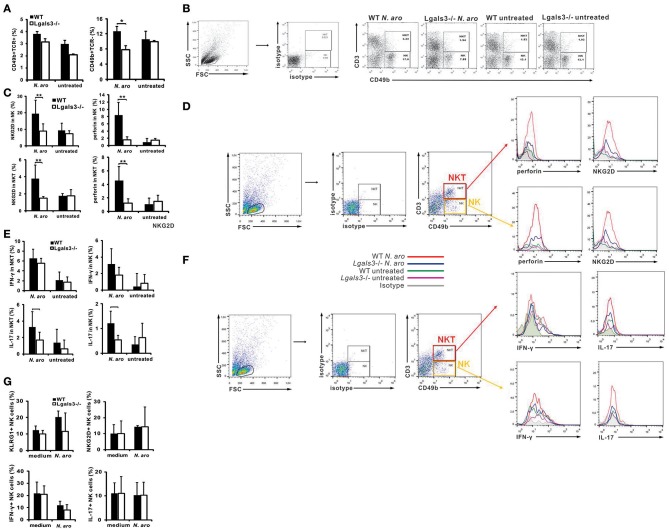
Gal-3 deficiency attenuates inflammatory and cytotoxic capacity of NK cells. Eight weeks after *N. aromaticivorans* infection mononuclear cells were isolated from livers and used for flow cytometric analysis of NK and NKT cells **(A)** Percentages of NK and NKT cells per liver. **(B)** Representative dot plots displaying the frequencies of NK and NKT cells in infected and untreated wild type and Lgals3^−/−^ mice. **(C)** Percentages of NKG2D and perforin positive cells in NK and NKT gated cells in liver in wild type and Gal-3 KO mice. **(D)** Expression of markers of activation and cytotoxicity on CD49b+CD3+ and CD49b+CD3- gated cells. **(E)** Percentages of IL-17 and IFN-γ positive cells in NK and NKT gated cells in the livers in WT and Lgals3^−/−^ mice. **(F)** Expression of IL-17 and IFN-γ on CD49b+CD3+ and CD49b+CD3- gated cells. Data are presented as mean+SE, 8 mice/group. NK cells were isolated from the livers of untreated WT and Lgals3^−/−^ mice (six mice in each group) cultured *in vitro* with *N. aromaticivorans* (cell/bacteria ratio 1:10) for 24 h and flow cytometric analysis was done. **(G)** Percentages of activated and IL-17 containing NK cells isolated from livers of untreated wild type and Lgals3^−/−^ mice and *in vitro* cultured with NA for 24 h., **p < 0.005; *p < 0.05.

**Figure 4:**
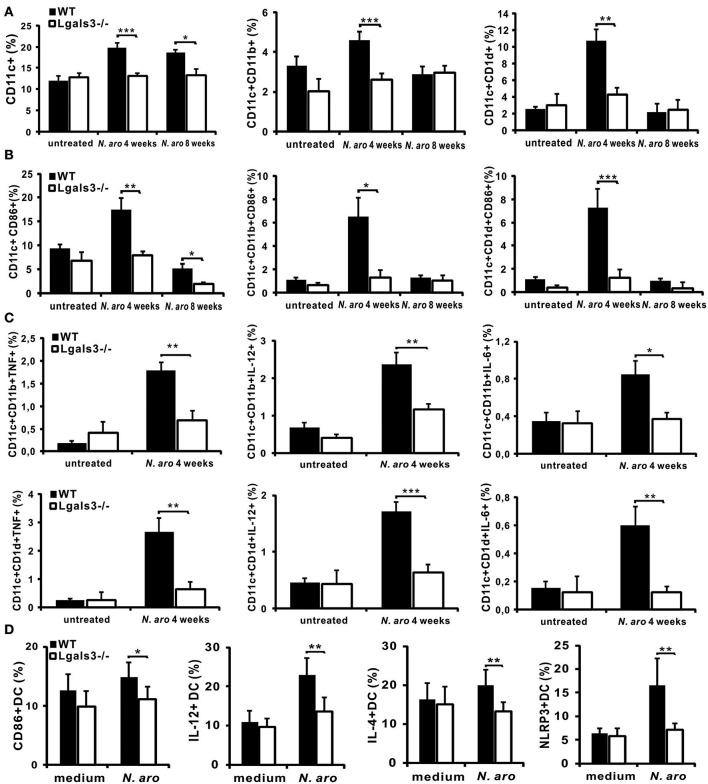
Gal-3 deficiency attenuates inflammatory DCs in liver. Four and eight weeks after *N. aromaticivorans* infection mononuclear cells were isolated from the livers and used for flow cytometric analysis of dendritic cells **(A)** Percentages of CD11c+, CD11c+CD11b+, and CD11c+CD1d+ dendritic cells per liver in WT and Lgals3^−/−^ mice. **(B)** Percentages of activated (CD86+) CD11c+, CD11c+CD11b+, and CD11c+CD1d+ dendritic cells per liver in WT and Lgals3^−/−^ mice. **(C)** Percentages of inflammatory (IL-12+, TNF-α, and IL-1β) CD11c+, CD11c+CD11b+, and CD11c+CD1d+ cells per liver in WT and Lgals3^−/−^ mice. Dendritic cells isolated from spleens of untreated WT and Lgals3^−/−^ mice, six mice per group, cultured *in vitro* with *N. aromaticivorans* (cell/bacteria ratio 1:10) for 24 h were analyzed for expression of markers of activation and cytokines using flow cytometry. **(D)** Percentages of CD86+, IL-12+, IL-4+, and NLRP3 expressing *N. aromaticivorans* stimulated WT and Lgals3^−/−^ dendritic cells. Data are presented as mean+SE, 8 mice in each group, ***p < 0.001,**p < 0.005; *p < 0.05.

Disease induction with *N. aromaticivorans* infection requires activity of NKT cells, activated with bacterium cell wall α-glycuronosylceramides presented by CD1d molecules (11). NK cells also contribute to activation of autoreactive T cells and enables cytopathic activity of these cells that contributes to biliary epithelial cell damage (16). Livers of *N. aromaticivorans* infected Lgals3^−/−^ mice contained significantly lower percentages of NK (CD49b+CD3-) cells (*p* < 0.05) compared with infected WT mice (Figures 3A,B). Moreover, infection didn't increase NK cell frequency in Lgals3^−/−^ mice. There was no significant difference in the percentage of NKT cells (CD49b+CD3+) between Lgals3^−/−^ and WT mice infected with *N. aromaticivorans* (Figures 3A,B). Therefore, we tested whether attenuated disease in Lgals3^−/−^ deficient mice was accompanied with decreased activation of NK and NKT cells. Significantly lower percentage of NK and NKT cells expressing markers of activation, NKG2D, and marker of cytotoxicity, perforin, was observed in the livers of *N. aromaticivorans* infected Lgals3^−/−^ mice (Figure 3C). Furthermore, NK and NKT cells of Lgals3^−/−^ mice had also lower expression of NKG2D and perforin (Figure 4D). No difference in percentage of NK and NKT cells containing IFN-γ was noticed between groups (Figure 3D). However, percentage of IL-17 positive NK and NKT cells was significantly lower in the liver of infected Lgals3^−/−^ mice (Figure 3E). NK and NKT cells in the livers of Lgals3^−/−^ *N. aromaticivorans* infected mice also had lower expression of IL-17 (Figure 3F).

In order to explore possible different direct effect of *N. aromaticivorans* on Lgals3^−/−^ and WT NK cells, these cells were isolated from the livers of untreated mice, incubated with bacteria for 24 h and expression of markers of activation and cytokines were analyzed by flow cytometry. No difference in expression of markers of activation (NKG2d and KLRG1) and inflammatory cytokines (IFN-γ and IL-17) in unstimulated and *N. aromaticivorans* stimulated NK cells, was found between two groups (Figure 3G).

### Galectin-3 Deficiency Attenuates Influx of Inflammatory Dendritic Cells in the Livers of Infected Mice

Next we analyzed the percentage of CD11c+, myeloid CD11c+CD11b+, and CD11c+CD1d+ dendritic cells in the livers of infected Lgals3^−/−^ and WT mice (Supplementary Figure 2). Four weeks after infection significantly lower percentage of CD11c+, CD11c+CD11b+, and CD11c+CD1d+ dendritic cells was detected in the livers of Lgals3^−/−^ mice compared with WT mice (Figure 4A). Eight weeks after infection this difference was significant only for CD11c+ cells. Also, 4 weeks after infection, significantly higher percentage of activated, CD86 positive, CD11c+, CD11c+CD11b+, and CD11c+CD1d+ dendritic cells was detected in the livers of WT compared with Lgals3^−/−^ mice (Figure 4B). Further, percentage of inflammatory TNFα+, IL-12+, and IL-6+ myeloid CD11c+CD11b+ and glycolipid presenting CD11c+CD1d+ cells was significantly lower in Lgals3^−/−^ mice compared with WT mice (Figure 4C). Dendritic cells isolated from spleens of untreated WT mice after *in vitro* stimulation with *N. aromaticivorans* had higher expression of marker of activation CD86 compared with Lgals3^−/−^ dendritic cells (Figure 4D). Further, *in vitro* stimulation with *N. aromaticivorans* significantly increased percentage of IL-12, IL-4, and NLRP3 positive WT dendritic cells in comparison with Lgals3^−/−^ dendritic cells (Figure 4D).

### Gal-3 Deficiency Significantly Attenuates Early Activation of Dendritic Cells With *N. aromaticivorans*

**Figure 5:**
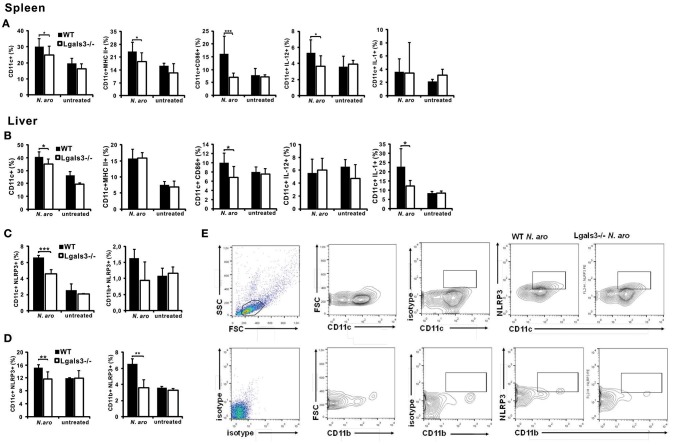
Gal-3 deficiency significantly attenuates activation of dendritic cells early after *N. aromaticivorans* infection, in spleen and liver. Three days after *N. aromaticivorans* infection mononuclear cells were isolated from the livers and spleens of WT and Lgals3^−/−^ mice and flow cytometric analysis was done. Percentages of CD11c+ dendritic cells, and CD11c+ dendritic cells expressing markers of activation and inflammatory cytokines per spleen **(A)** and liver **(B)**. Percentages of NLRP3+ dendritic and myeloid cells in spleen **(C)** and liver **(D)** in WT and Lgals3^−/−^ mice with representative dot plots **(E)**, 3 days after *N. aromaticivorans* infection. Data are presented as mean+SE, 8 mice/group, ***p < 0.001,**p < 0.005; *p < 0.05.

Having in mind that *N. aromaticivorans* can directly activate dendritic cells, and given the fact that place of initial immune response to *N. aromaticivorans* that leads to PBC, is not known, we analyzed phenotypes of these cells in both, spleen and liver, early after infection of Lgals3^−/−^ and WT mice. Three days after infection percentage of dendritic cells in the spleen (Figure 5A) and liver (Figure 5B) of Lgals3^−/−^ mice was significantly lower in comparison with WT infected mice. Spleens of WT mice, 3 days after *N. aromaticivorans* infection, contained significantly higher percentage of activated CD11c+CD86+, CD11c+MHCII+ and inflammatory IL-12+ dendritic cells in comparison with Lgals3^−/−^ mice (Figure 5A). Also, livers of infected WT mice contained significantly higher percentage of CD86+ and inflammatory IL-1β+ dendritic cells (Figure 5B).

Significant increase of NLRP3 inflammasome positive dendritic cells in the spleen (Figure 5C) and liver (Figure 5D) after infection was detected only in WT mice. Frequency of NLRP3+ dendritic cells was significantly higher both in the livers and spleens of WT infected mice compared with Lgals3^−/−^ infected mice (Figure 5E). Further, higher percentage of inflammasome positive cells was detected in the livers of WT infected mice than in the spleens.

### Gal-3 Deficiency Significantly Attenuates NLRP3 Inflammasome Expression, IL-1β Production, and caspase-1 Activity in *N. aromaticivorans* Stimulated Peritoneal Macrophages

**Figure 6:**
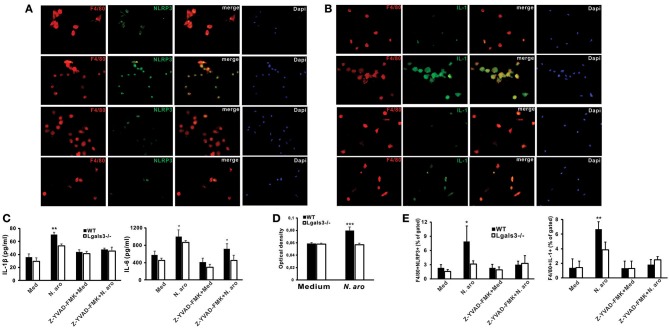
*Novosphyngobium aromaticivorans* stimulated Gal-3 deficient peritoneal macrophages have increased NLRP3 inflammasome expression and IL-β production. Immunofluorescence staining for F4/80 (red) and NLRP3 inflammasome **(A)**, or IL-1 **(B)** (green) with DNA staining with DAPI (blue) in peritoneal macrophages from wild type (upper two panels) and Lgals3^−/−^ mice (lower two panels) cultured for 7 days with *Novosphyngobium aromaticivorans* (first and third panel) or in medium only (second and forth panel). **(C)** Significantly higher production of IL-1β by WT vs. Gal-3 KO peritoneal macrophages upon simulation with *Novosphyngobium aromaticivorans* (cell: bacteria ratio 1:10), whereas the production was significantly reduced in the presence of the caspase-1 inhibitor Z-YVAD (10 mmol/L). Wild type macrophages stimulated with bacteria produce more IL-6 when compared with WT macrophages but the production was not affected by caspase-1 inhibitor Z-YVAD. **(D)** Significantly increased caspase-1 activity in cell lysates of wild type peritoneal macrophages stimulated with *Novosphyngobium aromaticivorans* in comparison with Lgals3^−/−^ peritoneal macrophages. **(E)** Significantly lower percentage of F4/80+ NLRP3+ and F4/80+IL-1β+ macrophages in Gal-3 KO vs. WT mice when stimulated with *Novosphyngobium aromaticivorans*. Data are presented as mean+SD, 5 mice/group, ***p < 0.001,**p < 0.005; *p < 0.05.

Next we wanted to estimate NLRP3 inflammasome and IL-1β expression in macrophages isolated from untreated WT and Lgals3^−/−^ mice *in vitro* stimulated with *N. aromaticivorans* (cell to bacteria ratio 1:10). Immunofluorescence staining showed that *N. aromaticivorans* induced increased NLRP3 inflammasome expression only in WT macrophages (Figure 6A, second panel), while there was no increase of NLRP3 expression in Lgals3^−/−^ macrophages (Figure 6A, fourth panel) in comparison with unstimulated macrophages (Figure 6A, first and third panels). Similarly, increase of IL-1β expression in *N. aromaticivorans* stimulated macrophages (Figure 6B, second panel) in comparison with unstimulated (Figure 6B, first panel) was observed only in WT macrophages. IL-1β expression in *N. aromaticivorans* stimulated Lgals3^−/−^ macrophages (Figure 6A, fourth panel) is as low as in unstimulated WT (Figure 6B, first panel) and Lgals3^−/−^ (Figure 6B, third panel) macrophages.

We showed the inherent genotype differences in the cellular response of WT vs. Lgals3^−/−^ mice to *N. aromaticivorans* as *in vitro* culture with this bacteria (cell to bacteria ratio 1:10) induced significantly higher production of IL-1β and IL-6 in WT peritoneal macrophages (Figure 6C). IL-1β production was significantly reduced in the presence of the caspase-1 inhibitor Z-YVAD-FMK (10 μmol/L), whereas IL-6 production was higher in WT macrophages even in the presence of Z-YVAD-FMK (Figure 6C). No significant difference in IL-1β production between Lgals3^−/−^ and WT mice was observed when cells were cultured in medium only. Stimulation of peritoneal macrophages with *N. aromaticivorans in vitro* significantly increased caspase-1 activity (Figure 6D) and significantly increased percentages of NLRP3 inflammasome- and IL-1β- expressing F4/80^+^ macrophages (Figure 6E) in WT compared with Lgals3^−/−^ macrophages.

### Galectin-3 Inhibitor Significantly Reduces Bile Duct Damage Induced by *N. aromaticivorans* Infection

**Figure 7:**
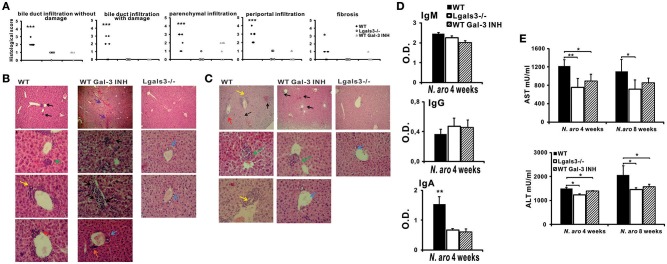
DAVANAT treatment attenuates PBC in C57BL/6 mice. C57BL/6 WT and Lgals3^−/−^ mice received two intraperitoneal injections of *N. aromaticivorans* (10^7^ CFU). One group of WT mice was treated with Gal-3 inhibitor from the first day of experiment (three times per week, in the course of 4 weeks). Four, eight and 24 weeks after *N. aromaticivorans* infection liver tissues were fixed, sectioned and stained with hematoxylin and eosin **(A)** Histological score for parameters of liver inflammation, granuloma formation, and fibrosis 8 weeks after infection presented per mouse. **(B)** Representative liver sections of WT, Lgals3^−/−^, and Gal-3 inhibitor treated WT mice, four (5 mice per group) and 24 (5 mice per group) weeks after *N. aromaticivorans* infection, arrows indicate: bile duct mononuclear cell infiltrate (yellow); parenchymal infiltration (black); organized parenchymal infiltration (green), parenchymal necrosis (violet), perivascular infiltrates (orange), bile duct obliteration (red); no infiltration (blue). **(D)** Serum levels of anti-PDC-E2 antibodies **(C)** in WT, Lgals3^−/−^, and Gal-3 inhibitor treated WT mice 4 weeks after infection with NA (5 mice per group). **(E)** Serum levels of AST and ALT in WT, Lgals3^−/−^, and Gal-3 inhibitor treated WT mice 4 (5 mice per group) and 8 weeks (5 mice per group) after infection with *N. aromaticivorans*. Data presented as mean + SE, *p < 0.05, **p < 0.005, ***p < 0.001.

After we have shown that Lgals3 deletion significantly reduces bile duct damage in *Novosphingobium aromaticivorans* infected mice, further experiments were done in order to determine the possible effect of Gal-3 inhibitor (DAVANAT^®^) on the disease. Treatment with inhibitor of Gal-3 in inductive phase of disease significantly reduced all parameters of PBC. Four weeks after *N. aromaticivorans* infection all histological parameters indicating liver and bile duct inflammation and fibrosis were significantly more pronounced in WT mice in comparison with Gal-3 inhibitor treated WT mice (Figure 7A). Four weeks after infection only mild infiltration was noticed in the livers of Lgals3^−/−^ mice, while intensive parenchymal and bile duct infiltrations and bile duct obliterations were observed in the livers of infected WT mice (Figure 7B). Interestingly and at variance what was seen in Lgals3^−/−^ mice, in the group of Gal-3 inhibitor treated WT mice mild parenchymal and perivascular infiltration and liver necrosis were detected (Figure 7B). However, 24 weeks after infection peribiliar infiltrates retained, and parenchymal granulomas were detected in the livers of infected WT mice (Figure 7C). In Gal-3 inhibitor treated mice 24 weeks after infection there was no longer necrosis, bile duct infiltration and damage in livers, only parenchymal granulomas (Figure 7C).

Four weeks after infection serum level of anti-PDC-E2 IgA in Gal-3 inhibitor treated mice was significantly lower in comparison with WT mice (Figure 7D). No difference in serum level of anti-PDC-E2 IgG and IgM was detected between all three groups of mice. Serum concentration of AST and ALT was significantly lower in Gal-3 inhibitor treated mice compared with WT mice, 4 weeks after infection (Figure 7E).

### NLRP3 Inflammasome and IL-1β Expression Are Significantly Lower in the Livers of *N. aromaticivorans* Infected Lgals3^−/−^ Mice and WT Mice Treated With Gal-3 Inhibitor

**Figure 8:**
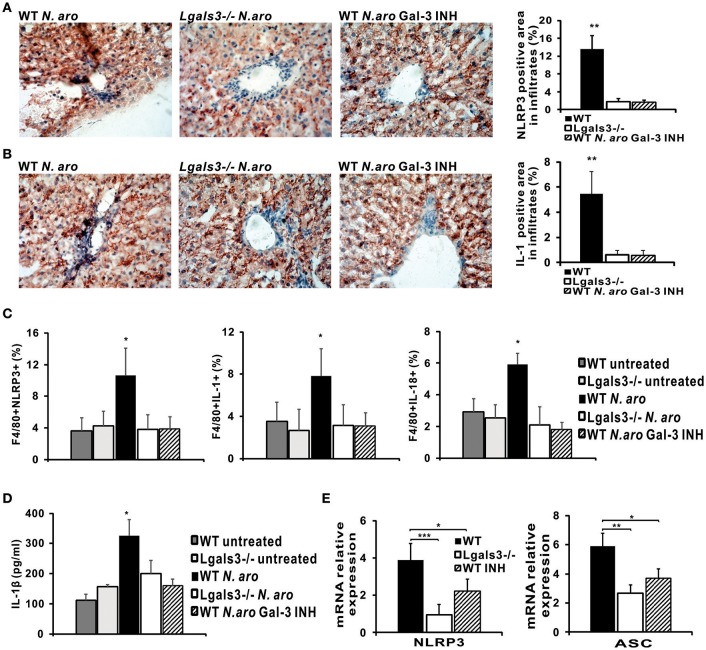
DAVANAT treatment decreases expression of NLRP3 inflammasome and IL-1β in liver infiltrates of *N. aromaticivorans* infected WT mice. Seven days after *N. aromaticivorans* infection of WT, Lgals3^−/−^ and Gal-3 inhibitor treated mice (7 or 8 mice per group) immunohistochemical staining of liver sections with analysis of the percentage of NLRP3 **(A)** and IL-1β **(B)** positive cells in liver infiltrates was done. **(C)** Percentages of F4/80 macrophages positive for NLRP3, IL-1β, and IL-18 in liver infiltrates 7 days after infection determined by flow cytometry. **(D)** IL-1β production determined by ELISA in liver tissue homogenate 7 days after infection. Data are presented as mean+SD. **(E)** NLRP3 and ASC inflammasome components expression in livers determined using real-time qRT-PCR, 15 weeks after infection, presented as mean+SE, *p < 0.05, **p < 0.005, ***p < 0.001.

To confirm lower expression of NLRP3 inflammasome and IL-1β production in Lgals3^−/−^ mice we next analyzed expression of these molecules in the liver infiltrates 7 days after *N. aromaticivorans* infection in WT, Lgals3^−/−^ and Gal-3 inhibitor treated WT mice. Immunohistochemistry data showed increased NLRP3 inflammasome expression in the liver infiltrates of infected WT mice compared with infected Lgals3^−/−^ and Gal-3 inhibitor treated WT mice (Figure 8A, Supplementary Figure 3) with a significantly higher percentage of NLRP3 inflammasome positive area in liver infiltrates of infected WT mice (Figure 8A). Also, increased IL-1β expression in liver infiltrates of infected WT mice compared with infected Lgals3^−/−^ and Gal-3 inhibitor treated WT mice was found, with a significantly higher percentage of IL-1β-positive area in liver infiltrates of infected WT mice (Figure 8B). Furthermore, significantly higher percentages of NLRP3 inflammasome, IL-1β, and IL-18 expressing F4/80+ macrophages was found in mononuclear cells derived from liver tissue of infected WT mice compared with infected Lgals3^−/−^ and Gal-3 inhibitor treated WT mice (Figure 8C, Supplementary Figure 4).

Further, ELISA analysis of liver tissue homogenates obtained from infected WT, Lgals3^−/−^, and Gal-3 inhibitor treated mice showed a significantly higher expression of IL-1β in infected WT mice compared with infected Lgals3^−/−^ and Gal-3 inhibitor treated and uninfected, control mice (Figure 8D). PCR analysis showed a significantly higher relative expression of inflammasome components, ASC and NLRP3, in the livers of infected WT mice compared with infected Lgals3^−/−^ and Gal-3 inhibitor treated WT mice (Figure 8E).

**Figure 9:**
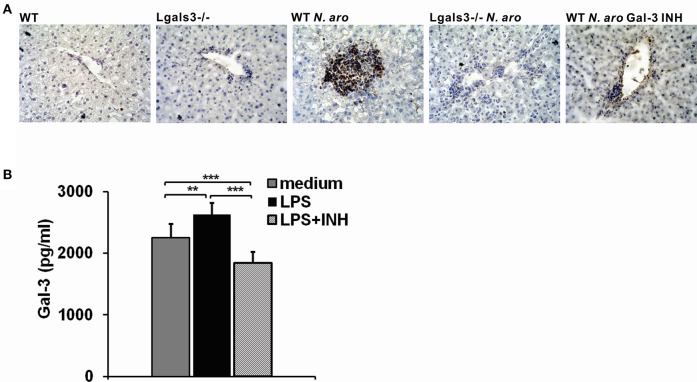
DAVANAT treatment decreases Gal-3 expression in liver infiltrates of *N. aromaticivorans* infected WT mice. **(A)** Representative sections of Gal-3 immunohistochemical staining in liver sections obtained from WT, Lgals3^−/−^ and Gal-3 inhibitor treated mice 7 days after *N. aromaticivorans* infection. **(B)** Gal-3 levels in cell culture supernatants of splenocytes obtained from WT mice, stimulated *in vitro* with LPS (1 μg/ML) for 24 h, or treated with Gal-3 inhibitor (100 μM) for 2 h before LPS stimulation, or left untreated, presented as mean+SD, **p < 0.005, ***p < 0.001.

Immunohistochemistry of liver sections showed no Gal-3 expression in untreated control mice, while significant increase in Gal-3 expression was detected in the liver infiltrates in WT mice seven days after *N. aromaticivorans* infection (Figure 9A). There was no Gal-3 expression in the livers of both untreated and infected Lgals3^−/−^ mice (Figure 9A). Also there was negliglable Gal-3 expression in the liver infiltrates of Gal-3 inhibitor treated WT mice (Figure 9A). To confirm that Gal-3 inhibitor reduces Gal-3 levels, splenocytes isolated from WT mice were treated with DAVANAT® before LPS stimulation, and Gal-3 was measured in cell culture supernatants using ELISA. *In vitro* treatment of mouse splenocytes with Gal-3 inhibitor before LPS priming significantly reduced concentration of Gal-3 in cell culture supernatants (Figure 9B).

### Gal-3 Inhibitor Significantly Attenuates Influx of Type1, Type 2, and Type 17 Lymphocytes in the Liver of *N. aromaticivorans* Infected Mice

*N. aromaticivorans* infection did not induce significant increase of total number of analyzed populations of lymphocytes in Lgals3^−/−^ and Gal-3 inhibitor treated WT mice (Table 1). Four weeks after *N. aromaticivorans* infection significantly higher frequencies of Tbet, RORγt, and IL-17 expressing CD4+ and CD8+ cells and CD4+IFN-γ+ cells were detected in the livers of WT mice compared with Lgals3^−/−^ and Gal-3 inhibitor treated WT mice and uninfected Lgals3^−/−^ and WT mice (Table 1). Total numbers of CD4+ and CD8+ cells expressing type 1 (Tbet and IFN-γ), type 17 (RORγt, and IL-17), and type 2 (GATA3, IL-4, IL5, and IL-13) transcriptional factors and cytokines, except CD8+IL-5+ cells, were significantly higher in the livers of *N. aromaticivorans* infected WT mice compared with infected Lgals3^−/−^ and Gal-3 inhibitor treated WT mice and untreated Lgals3^−/−^ and WT mice (Table 1). There was no increase in total number of these populations in the livers of infected Lgals3^−/−^ and WT mice treated with Gal-3 inhibitor compared with untretaed Lgals3^−/−^ and WT mice. There was no difference in the percentage and total number of Tregs (Foxp3 and IL-10 positive) between all three groups of infected mice and between *N. aromaticivorans* infected and untreated, control mice (Table 1).

**Table 1:** Phenotype Difference of CD4+ and CD8+ cells in the liver of WT.

	**Percentages**					**Total number** **×** **10**^****4****^				
	**WT untreated (*n* = 8)**	**LGASL3^**−/−**^ untreated (*n* = 8)**	**WT N. aro (*n* = 8)**	**LGASL3^**−/−**^ N. aro (*n* = 8)**	**WT N. aro Gal-3 inh (*n* = 8)**	**WT untreated (*n* = 8)**	**LGASL3^**−/−**^ untreated (*n* = 8)**	**WT N. aro (*n* = 8)**	**LGASL3^**−/−**^ N. aro (*n* = 8)**	**WT N. aro Gal-3 inh (*n* = 8)**
CD4+Tbet+	1.19 ± 0.76	2.05 ± 0.51	8.16 ± 2.42^*^	3.41 ± 0.75	2.68 ± 0.49	6.64 ± 4.91	9.20 ± 2.00	96.27 ± 38.84^**^	17.51 ± 8.17	8.53 ± 4.55
CD4+IFN-γ+	0.82 ± 0.53	0.77 ± 0.63	5.49 ± 1.88^*^	1.74 ± 0.96	0.92 ± 0.62	4.53 ± 3.02	3.42 ± 2.92	62.68 ± 22.68^**^	8.65 ± 5.05	3.18 ± 2.94
CD8+Tbet+	0.51 ± 0.22	1.09 ± 0.15	4.05 ± 0.82^*^	1.23 ± 0.24	1.89 ± 0.80	2.77 ± 1.38	5.01 ± 1.18	46.47 ± 10.58^*^	6.40 ± 3.08	5.40 ± 2.34
CD8+IFN-γ+	1.10 ± 0.88	0.99 ± 0.96	3.41 ± 1.68	1.76 ± 1.06	1.15 ± 0.89	5.99 ± 4.88	4.29 ± 4.27	39.59 ± 21.08^*^	8.69 ± 5.46	3.99 ± 4.22
CD4+RORγt+	1.48 ± 0.57	1.39 ± 0.48	3.49 ± 0.62^*^	2.08 ± 0.33	1.92 ± 0.65	8.33 ± 4.39	6.66 ± 2.91	41.69 ± 11.09^*^	18.44 ± 3.18	6.77 ± 4.5
CD4+IL-17+	1.09 ± 0.81	1.70 ± 0.87	7.14 ± 2.08^*^	2.65 ± 0.55	1.42 ± 0.46	6.06 ± 5.13	7.88 ± 4.43	82.36 ± 21.57^***^	11.15 ± 4.39	4.74 ± 3.01
CD8+RORγt+	1.07 ± 0.29	0.82 ± 0.23	2.57 ± 0.34^**^	1.46 ± 0.23	0.96 ± 0.29	6.00 ± 2.55	3.74 ± 1.20	30.05 ± 1.94^**^	12.94 ± 2.35	3.10 ± 1.34
CD8+IL-17+	1.24 ± 0.55	1.73 ± 0.70	5.01 ± 1.31^*^	1.52 ± 0.49	1.76 ± 0.31	7.00 ± 3.84	8.04 ± 3.60	58.24 ± 21.19^**^	7.96 ± 4.74	5.67 ± 2.99
CD4+GATA3+	1.33 ± 0.41	1.65 ± 0.64	2.66 ± 0.81	1.89 ± 0.48	1.54 ± 0.55	7.41 ± 3.27	7.16 ± 1.39	31.42 ± 13.22^*^	9.93 ± 5.50	4.54 ± 1.78
CD4+IL-4+	1.24 ± 0.71	1.66 ± 0.40	3.17 ± 0.87	1.66 ± 0.57	1.89 ± 0.71	7.10 ± 4.75	7.83 ± 2.73	36.71 ± 11.72^*^	8.29 ± 3.55	6.02 ± 3.30
CD4+IL-5+	1.08 ± 0.94	1.26 ± 1.48	4.15 ± 2.17	1.24 ± 0.73	1.21 ± 0.86	6.96 ± 6.90	6.02 ± 7.52	49.63 ± 32.17^*^	5.51 ± 2.58	4.31 ± 3.92
CD4+IL-13+	1.08 ± 0.94	1.69 ± 0.63	3.38 ± 0.88	1.73 ± 0.55	1.49 ± 0.62	6.07 ± 3.68	7.99 ± 3.56	38.81 ± 12.04^*^	8.75 ± 4.50	4.90 ± 3.27
CD8+GATA3+	0.87 ± 0.49	0.85 ± 0.47	1.06 ± 0.37	0.69 ± 0.18	0.84 ± 0.27	4.74 ± 2.97	3.89 ± 2.45	12.15 ± 4.38^*^	3.56 ± 1.96	2.50 ± 1.05
CD8+IL-4+	0.65 ± 0.32	0.98 ± 0.59	1.19 ± 0.36	0.61 ± 0.28	0.80 ± 0.34	3.68 ± 2.18	4.63 ± 2.79	13.45 ± 3.25^*^	3.18 ± 2.07	2.50 ± 1.40
CD8+IL-5+	0.36 ± 0.40	0.46 ± 0.77	0.77 ± 0.96	0.31 ± 0.31	0.48 ± 0.53	2.41 ± 2.97	2.23 ± 3.88	9.65 ± 12.94	1.27 ± 1.19	1.74 ± 2.06
CD8+IL-13+	0.83 ± 0.27	1.04 ± 0.33	1.89 ± 0.36	0.96 ± 0.36	1.00 ± 0.16	4.72 ± 2.12	4.99 ± 2.26	21.98 ± 6.22^*^	4.89 ± 2.59	2.60 ± 1.90
CD4+Foxp3+	1.04 ± 0.72	1.82 ± 1.48	1.96 ± 0.64	1.61 ± 1.16	1.88 ± 1.35	6.15 ± 4.94	8.69 ± 8.83	23.23 ± 7.72	12.71 ± 5.74	6.22 ± 4.74
CD4+IL-10+	1.30 ± 0.81	2.47 ± 0.50	1.91 ± 1.51	0.89 ± 0.65	1.36 ± 1.56	7.68 ± 5.37	11.12 ± 2.19	22.14 ± 17.84	4.17 ± 3.26	5.14 ± 6.72
CD8+Foxp3+	0.35 ± 0.31	0.69 ± 1.07	0.65 ± 0.58	0.55 ± 0.77	0.71 ± 1.00	2.13 ± 2.06	3.34 ± 5.39	7.30 ± 6.09	4.14 ± 3.49	2.10 ± 2.89
CD8+IL-10+	1.51 ± 0.75	1.51 ± 0.59	1.67 ± 1.74	0.66 ± 0.43	1.77 ± 2.21	8.83 ± 5.22	11.64 ± 7.19	21.22 ± 24.26	3.10 ± 2.01	6.80 ± 9.66

*Lgals3^−/−^ and Gal-3 inhibitor treated WT mice four weeks after disease induction*.

*The data are presented as means ± SD*.

* *significantly different (p < 0.05) WT N. aro vs. Lgasl3^−/−^ N. aro and WT N. aro Gal-3 inh*.

** *significantly different (p < 0.005) WT N. aro vs. Lgasl3^−/−^ N. aro and WT N. aro Gal-3 inh*.

*** *significantly different (p < 0.001) WT N. aro vs. Lgasl3^−/−^ N. aro and WT N. aro Gal-3 inh. Statistical significance was tested by ANOVA*.

### Gal-3 Inhibitor Treatment Significantly Reduces Liver Fibrosis in *N. aromaticivorans* Infected Mice

**Figure 10:**
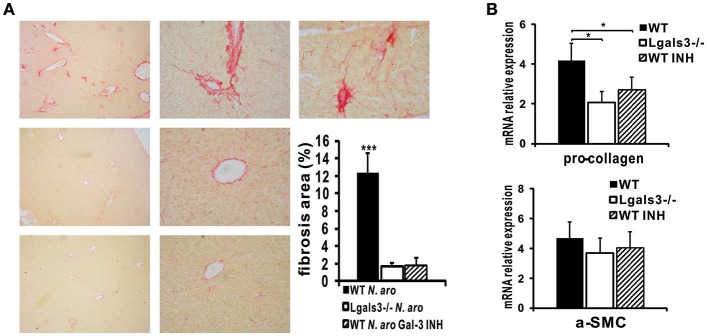
DAVANAT treatment decreases liver fibrosis *N. aromaticivorans* induced cholangitis. **(A)** Representative images of Picrosirius staining and quantitaive analysis of fibrosis in liver sections obtained from WT, Lgals3^−/−^ and Gal-3 inhibitor treated mice 3 months after infection, indicating negliglable fibrosis in Lgals3^−/−^ and Gal-3 inhibitor treated WT mice and marked fibrosis in WT mice. **(B)** Pro-collagen and α-SMC expression in livers determined using real-time qRT-PCR, 15 weeks after infection. Data are presented as mean+SE, *p < 0.05, ***p < 0.001.

Picrosirius red staining of liver sections obtained 3 months after *N. aromaticivorans* infection demonstrated liver fibrosis in WT mice (Figure 10A, top panel). Negligible deposit of collagen was observed in Lgals3^−/−^ (Figure 10A, middle panel) and Gal-3 inhibitor treated (Figure 10A, bottom panel) mice 3 months after infection. Percentage of area with deposited collagen was significantly higher in the livers of infected WT mice compared with Lgals3^−/−^ and Gal-3 inhibitor treated WT mice (Figure 10A). Also, Lgals3^−/−^ and Gal-3 inhibitor treated WT mice had significantly reduced expression of pro-fibrotic molecule pro-collagen in the liver, compared with WT mice (Figure 10B). Lower expression of pro-fibrotic α-SMC has been noticed in the livers of Lgals3^−/−^ and Gal-3 inhibitor treated WT mice compared with WT mice, but the difference did not reach statistical significance. No significant difference in the expression of pro-fibrotic molecules was noticed between Gal-3 deficient and Gal-3 inhibitor treated WT mice (Figure 10B).

## Discussion

Here, we have provided the evidence that targeted disruption of Gal-3 significantly attenuates autoimmune cholangitis in C57BL/6 mice induced by *N. aromaticivorans* infection. Attenuated bile duct damage in *N. aromaticivorans* infected Lgals3^−/−^ mice is accompanied with lower serum level of anti-PDC-E2 IgA, AST, and ALT, attenuated type 17 immune response in liver, and attenuated activation of inflammasome, in the liver and spleen. Additionally, we have provided the first evidence that pharmacological inhibition of Gal-3 with DAVANAT^®^ attenuated autoimmune cholangitis, suppressed serum levels of anti-PDC-E2 IgA, AST and ALT, expression of NLRP3 inflammasome, IL-1β production and influx of T1, T2, and T17 cells in the liver, and reduced liver fibrosis.

Recent study indicates that physiological effects of DAVANAT^®^ are not mediated by its inhibition of the canonical carbohydrate-binding site of Gal-3 (17) and questions its selectivity. Further, in cell-based assays, indirectly linked to galectin-3 inhibition, Stegmayr et al did not show inhibition of Gal-3 by DAVANAT^®^ (17). Miller et al. showed that DAVANAT^®^ interacts primarily with a non-canonical carbohydrate-binding site on the F-face of the Gal-3 (18). However, short time treatment with DAVANAT^**®**^ of tumor infiltrating lymphocytes (TIL) isolated from human tumor ascites boosts cytotoxicity of CD8+ TILs and their IFN-γ secretion in a dose dependent manner (16). Further, several studies indicate the role of DAVANAT^®^ *in vivo* in modulation of various inflammatory diseases. Lower liver inflammation and fibrosis in animals treated with DAVANAT^®^ in mouse model of non-alcoholic fatty liver and thioacetamide-induced liver disease were previously reported (19, 20). Treatment of mice with dextran sulfate sodium (DSS) induced colitis with mesenchymal stem cells cultured in the presence of DAVANAT^®^ decreased concentration of Gal-3 in sera of these animals (21), while treatment of mice with DAVANAT^®^ in the time of induction of DSS colitis attenuated the disease (22). Our findings of attenuation of Gal-3 expression in liver infiltrates (Figure 9A) in *N. aromaticivorans* infected Gal-3 inhibitor treated mice and reduced concentration of Gal-3 in supernatants of cell culture *in vitro* treated with Gal-3 inhibitor (Figure 9B) indicate that DAVANAT®, although does not bind to canonical carbohydrate-binding site of Gal-3, inhibits Gal-3 expression.

Mouse model of autoimmune cholangitis induced with *N. aromaticivorans* infection is, in comparison with xenobiotic induced disease, more similar to natural history of PBC in humans, since it is known that gut microbiota contributes to a mouse model of spontaneous bile duct inflammation (23). *N. aromaticivorans*, ubiquitous α-proteobacterium, can be detected in human mucous membranes of digestive system, contains conserved epitope PDC-E2, metabolizes xenobiotics and interferes with enterohepatic circulation of bile acids (24). We have shown that deletion of Gal-3 attenuates *N. aromaticivorans* induced PBC by affecting innate and acquired immune response (Figures 2–8). Cell wall of *N. aromaticivorans* contains glysphingolipids that activate NKT cells presented in CD1d complex on dendritic cells (12). Cytokines secreted by activated antigen presenting cells, such as myeloid dendritic cells and Kupffer cells, contribute to NKT activation (25). Activated NKT cells release cytokines that stimulate further dendritic cell activation and thus have critical role in antimicrobial immunity toward *N. aromaticivorans*, but also in activation of autoreactive helper T cells (13, 26). The presence of NKT and CD1d expressing dendritic cells is significantly increased in the livers of PBC patients (27–29). We observed a lower proportion of NKT, CD1d+, and CD11b+ dendritic cells in the livers of Lgals3^−/−^ mice that almost did not develop PBC (Figures 3, 4).

The key elements for PBC pathology, small bile duct infiltrations and granuloma formation, are detected in the livers of infected C57BL/6 mice, as it was shown in the initial report of *N. aromaticivorans* induced cholangitis in NOD 1101 mice (11). In Lgals3^−/−^ mice only mild bile duct infiltration without damage was observed (Figure 1). Unlike the finding reported in NOD 1101 mice, we detected liver fibrosis in infected C57BL/6 mice (Figures 1, 7, 10). Production of Th2 cytokines is considered indispensable for fibrosis development (30). Th2 immune response is significantly attenuated in NOD mice (31), while C57BL/6 WT mice, although less than BALB/c mice, still produce Th2 cytokines (32). Dendritic cells isolated from WT mice *in vitro* stimulated with *N. aromaticivorans* produce IL-4 (Figure 4D), cytokine needed for Th2 differentiation. Also, significant influx of CD4+ and CD8+ cells expressing pro-fibrotic cytokines, IL-4 and IL-13, was noticed in the livers of infected WT mice (Table 1).

Although PBC is generally considered as Th1 mediated autoimmune disease, there are data indicating significant role of Th17 cells in its initiation and progression. Significant accumulation of Th17 cells and high expression of CCL20 has been described in livers of PBC patients (33). The other study indicates significant presence of IL23+ and IL-17+ mononuclear cells in portal tracts in late stages of PBC (34). Livers of IL-2Rα knockout mice that spontaneously develop PBC-like disease contain aggregates of IL-17+ lymphocytes in portal tracts (15). Presence of IL-17+ lymphocytes in the liver is typical for later stages of PBC, indicating significance of Th1 to Th17 conversion for disease progression (35).

In accordance with histological and serum parameters of disease, significantly lower percentages of IL-17+ cells was found in the livers of infected Lgals3^−/−^ mice (Figure 2). Significantly reduced influx of T1, T17, and T2 cells was noticed in the livers of Gal-3 inhibitor treated mice (Table 1). Among inflammatory lymphocytes in the livers of infected WT mice is the highest total number of IL-17 expressing CD4+ and CD8+ cells (Table 1). Taking into account our results and previous reports (35) it can be assumed that the main role in development of *N. aromaticivorans* induced PBC in C57BL/6 mice play IL-17 producing lymphocytes. Also, higher expression of IL-17 in NK and NKT cells in infected C57BL/6 mice that develop disease found in our study (Figure 3) are in line with previous finding that liver microenvironment potentiate IL-17 production (15).

Phenotyping of dendritic cells in the livers and spleens 3 days after *N. aromaticivorans* infection showed significantly lower percentage of activated and IL-12+ dendritic cells in the spleens and lower percentage of IL-1+ dendritic cells in the livers of Lgals3^−/−^ mice in comparison with WT mice (Figure 5). Further, in the phase of developed disease, significantly lower percentage of activated and inflammatory myeloid CD11b+ and lipid presenting CD1d+ dendritic cells was detected in the livers of Lgals3^−/−^ mice (Figure 4). These results are in contrast with our results obtained in xenobiotic induced PBC, where the expression of Gal-3 in biliary epithelial cells is most important for observed effect (9), but are in accordance with previous reports that downregulation of Gal-3 in dendritic cells inhibits production of inflammatory cytokines (36). Significantly lower percentage of IL-1+ dendritic cells in the liver of Lgals3^−/−^ mice with no difference in percentage of IL-12+ dendritic cells (Figure 5) could explain reduced percentage of CD4+17+ and CD8+IL17+ cells in the livers of these mice. Attenuation of inflammatory dendritic cells in the abscence of Gal-3 is in accordance with studies about the role of Gal-3 in pathogenesis of other inflammatory liver diseases (37–39) and acute colitis (22).

There is no data about possible interaction of Gal-3 and *Novosphingobium aromaticivorans*. However, taking into account interaction of Gal-3 and different bacterial glycoconjugates and glycosphingolipides on human cells (40), it is very likely that Gal-3 can directly bind glycosphingolipides of *N. aromaticivorans*. Related to this assumption, stimulation *in vitro* of dendritic cells isolated from healthy WT mice with *N. aromaticivorans* results in increased expression of marker of activation and inflammatory cytokines (Figure 4D). *N. aromaticivorans* stimulation of Gal-3 deficient dendritic cells did not induce this effect. These results indicate that Lgals3^−/−^ mice do not develop disease after *N. aromaticivorans* infection most likely due to inability of dendritic cells to be activated with *N. aromaticivorans* in the abscence of Gal-3 hindering adequate activation of NKT and autoreactive T cells. This conclusion is in accordance with previous reports that Gal-3 enhances capacity of dendritic cells to stimulate effector function of NKT cells and liver damage in α-galactosilceramide induced hepatitis (39).

Activation of inflammasome in liver macrophages play significant role in pathogenesis of liver diseases (41). Transgenic mice that constitutively express active NLRP3 have significant pyroptosis of hepatocytes, inflammation and fibrosis (41). Gal-3 is required for NLRP3 activation in macrophages and results in IL-17 responses *via* an autocrine mechanism (10). Significantly higher percentage of NLRP3+ dendritic cells and macrophages, higher production of IL-1β and higher expression of NLRP3 and ASC in the liver, early after *N. aromaticivorans* infection, were found in the livers of WT mice compared with Lgals3^−/−^ mice (Figures 5, 8). Further *in vitro* stimulation of dendritic cells with *N. aromaticivorans* significantly increases NLRP3 expression in Gal-3+ cells (Figure 5D), while *in vitro* stimulation of WT peritoneal macrophages with this bacteria results in increased expression of NLRP3 inflammasome, increased production of IL-1β and increased caspase-1 activity (Figure 6). These results are in line with previous report that Gal-3 deletion in dnTGF-βRII mice impairs inflammasome activation, attenuates Th17 immune response and significantly improves cholangitis (10).

Inflammasome activation has important role in pathogenesis of metabolic and inflammatory liver diseases triggered by repetitive weak stimuli. On the other hand strong stimulators of immune system in autoimmune hepatitis activates receptors of innate immunity (42). Different ways of stimulation of innate immune cells in xenobiotic and *N. aromaticivorans* induced PBC could explain the opposite effect of Gal-3 in these two models of PBC. *N. aromaticivorans* has atypical cell wall with glycosphingolipids similar to molecules in eukaryotic membranes that mostly does not induce inflammation and tissue damage, although it is detected on mucous membranes of the digestive tract (14). Thus, it can be assumed that activation of innate immunity and inflammatory response to *N. aromaticivorans* in mice are triggered by inflammasome activation (by integration of weak signals) resulting in further immune cell activation and cholangitis development. Absence of disease in Lgals3^−/−^ mice and significantly attenuated disease in Gal-3 inhibitor treated WT mice are in accordance with previous data on the role of Gal-3 in inflammasome activation (10, 22). In xenobiotic induced PBC dendritic cells are mainly activated by Freund's complete adjuvant that stimulates TLRs (43) diminishing the role of inflammasome in activation of innate immunity. It is possible that WT and Gal-3 deficient dendritic cells are similarly activated with mixture of xenobiotic and adjuvant, thus reducing the significance of Gal-3 in the initial phase of the disease. Also, based on our finding of increased inflammatory phenotype of dendritic cells in Gal-3 deficient mice immunized with xenobiotic and adjuvant (9) and known role of Gal-3 in attenuation of TLR agonist induced inflammation (43, 44) it can be assumed that Gal-3 attenuates activation of dendritic cells with mixture of xenobiotic and adjuvant and thus contributes to attenuation of xenobiotic induced PBC.

In conclusion, we propose that Gal-3 plays an important pro-inflammatory role in *N. aromaticivorans* induced autoimmune cholangitis probably due to dominant role of inflammasome in *N. aromaticivorans* induced activation of dendritic cells and macrophages, resulting in activation of other players in PBC pathogenesis, in particular IL-17 containing NK, NKT, and T cells. Inhibition of Gal-3 signaling may be a potential therapeutic strategy for primary biliary cholangitis.

## Ethics Statement

All experiments were approved by and conducted in accordance with the Guidelines of the Animal Ethics Committee of Faculty of Medical Sciences, University of Kragujevac, Serbia.

## Author Contributions

AA, MM, NA, and ML: experimental design. AA, JM, BS, DD, NJ, and IS: data acquisition and analyses. AA, MM, MJ, NA, DV, and ML: data interpretation. MM and AA: study concept and design. AA, MM, and ML: manuscript writing and critical revision. All authors approved the final version.

### Conflict of Interest Statement

The authors declare that the research was conducted in the absence of any commercial or financial relationships that could be construed as a potential conflict of interest.

## References

[1] Kaplan MM Gershwin ME. Primary biliary cirrhosis. N Engl J Med. (2005) 353:1261–73. 10.1056/NEJMra043898 16177252

[2] Zhang J Zhang W Leung PS Bowlus CL Dhaliwal S Coppel RL. Ongoing activation of autoantigen-specific B cells in primary biliary cirrhosis. Hepatology. (2014) 60:1708–16. 10.1002/hep.27313 25043065 PMC4211937

[3] Lleo A Selmi C Invernizzi P Podda M Coppel RL Mackay IR. Apotopes and the biliary specificity of primary biliary cirrhosis. Hepatology. (2009) 49:871–9. 10.1002/hep.22736 19185000 PMC2665925

[4] Shimoda S Harada K Niiro H Shirabe K Taketomi A Maehara Y. Interaction between Toll-like receptors and natural killer cells in the destruction of bile ducts in primary biliary cirrhosis. Hepatology. (2011) 53:1270–81. 10.1002/hep.24194 21400555 PMC3077894

[5] Wang L Sun Y Zhang Z Jia Y Zou Z Ding J. CXCR5 CD4 T follicular helper cells participate in the pathogenesis of primary biliary cirrhosis. Hepatology. (2015) 61:627–38. 10.1002/hep.27306 25042122 PMC4507804

[6] Sciacchitano S Lavra L Morgante A Ulivieri A Magi F De Francesco GP. Galectin-3: one molecule for an alphabet of diseases, from A to Z. Int J Mol Sci. (2018) 19:E379. 10.3390/ijms19020379 29373564 PMC5855601

[7] Radosavljevic G Volarevic V Jovanovic I Milovanovic M Pejnovic N Arsenijevic N. The roles of Galectin-3 in autoimmunity and tumor progression. Immunol Res. (2012) 52:100–10. 10.1007/s12026-012-8286-6 22418727

[8] de Oliveira FL Gatto M Bassi N Luisetto R Ghirardello A Punzi L. Galectin-3 in autoimmunity and autoimmune diseases. Exp Biol Med. (2015) 240:1019–28. 10.1177/1535370215593826 26142116 PMC4935288

[9] Arsenijevic A Milovanovic M Milovanovic J Stojanovic B Zdravkovic N Leung PS. Deletion of Galectin-3 enhances xenobiotic induced murine primary biliary cholangitis by facilitating apoptosis of BECs and release of autoantigens. Sci Rep. (2016) 6:23348. 10.1038/srep23348 26996208 PMC4800400

[10] Tian J Yang G Chen HY Hsu DK Tomilov A Olson KA. Galectin-3 regulates inflammasome activation in cholestatic liver injury. FASEB J. (2016) 30:4202–13. 10.1096/fj.201600392RR 27630169 PMC5102125

[11] Mattner J Savage PB Leung P Oertelt SS Wang V Trivedi O. Liver autoimmunity triggered by microbial activation of natural killer T cells. Cell Host Microbe. (2008) 3:304–15. 10.1016/j.chom.2008.03.009 18474357 PMC2453520

[12] Kawahara K Moll H Knirel YA Seydel U Zähringer U. Structural analysis of two glycosphingolipids from the lipopolysaccharide-lacking bacterium Sphingomonas capsulata. Eur J Biochem. (2000) 267:1837–46. 10.1046/j.1432-1327.2000.01189.x 10712617

[13] Kinjo Y Wu D Kim G Xing GW Poles MA Ho DD. Recognition of bacterial glycosphingolipids by natural killer T cells. Nature. (2005) 434:520–5. 10.1038/nature03407 15791257

[14] Kawata K Tsuda M Yang GX Zhang W Tanaka H Tsuneyama K. Identification of potential cytokine pathways for therapeutic intervention in murine primary biliary cirrhosis. PLoS ONE. (2013) 8:e74225. 10.1371/journal.pone.0074225 24040208 PMC3769355

[15] Lan RY Salunga TL Tsuneyama K Lian ZX Yang GX Hsu W. Hepatic IL-17 responses in human and murine primary biliary cirrhosis. J Autoimmun. (2009) 32:43–51. 10.1016/j.jaut.2008.11.001 19101114 PMC3225053

[16] Demotte N Bigirimana R Wieërs G Stroobant V Squifflet JL Carrasco J. A short treatment with galactomannan GM-CT-01 corrects the functions of freshly isolated human tumor-infiltrating lymphocytes. Clin Cancer Res. (2014) 20:1823–33. 10.1158/1078-0432.CCR-13-2459 24526733

[17] Stegmayr J Lepur A Kahl-Knutson B Aguilar-Moncayo M Klyosov AA Field RA. Low or no inhibitory potency of the canonical galectin carbohydrate-binding site by pectins and galactomannans. J Biol Chem. (2016) 291:13318–34. 10.1074/jbc.M116.721464 27129206 PMC4933242

[18] Miller MC Ippel H Suylen D Klyosov AA Traber PG Hackeng T. Binding of polysaccharides to human galectin-3 at a noncanonical site in its carbohydrate recognition domain. Glycobiology. (2016) 26:88–99. 10.1093/glycob/cwv073 26646771 PMC4851716

[19] Traber PG Chou H Zomer E Hong F Klyosov A Fiel MI. Regression of fibrosis and reversal of cirrhosis in rats by galectin inhibitors in thioacetamide-induced liver disease. PLoS ONE. (2013) 8:e75361. 10.1371/journal.pone.0075361 24130706 PMC3793988

[20] Traber PG Zomer E. Therapy of experimental NASH and fibrosis with galectin inhibitors. PLoS ONE. (2013) 8:e83481. 10.1371/journal.pone.0083481 24367597 PMC3867460

[21] Simovic Markovic B Nikolic A Gazdic M Nurkovic J Djordjevic I Arsenijevic N. Pharmacological Inhibition of Gal-3 in mesenchymal stem cells enhances their capacity to promote alternative activation of macrophages in dextran sulphate sodium-induced colitis. Stem Cells Int. (2016) 2016:2640746. 10.1155/2016/2640746 27057168 PMC4736319

[22] Simovic Markovic B Nikolic A Gazdic M Bojic S Vucicevic L Kosic M. Galectin-3 Plays an important pro-inflammatory role in the induction phase of acute colitis by promoting activation of NLRP3 inflammasome and production of IL-1β in macrophages. J Crohns Colitis. (2016) 10:593–606. 10.1093/ecco-jcc/jjw013 26786981 PMC4957458

[23] Schrumpf E Kummen M Valestrand L Greiner TU Holm K Arulampalam V. The gut microbiota contributes to a mouse model of spontaneous bile duct inflammation. J Hepatol. (2017) 66:382–9. 10.1016/j.jhep.2016.09.020 27720803 PMC5250551

[24] Selmi C Balkwill DL Invernizzi P Ansari AA Coppel RL Podda M. Patients with primary biliary cirrhosis react against a ubiquitous xenobiotic-metabolizing bacterium. Hepatology. (2003) 38:1250–7. 10.1053/jhep.2003.50446 14578864

[25] Kronenberg M Rudensky A. Regulation of immunity by self-reactive T cells. Nature. (2005) 435:598–604. 10.1038/nature03725 15931212

[26] Mattner J Debord KL Ismail N Goff RD Cantu C Zhou D. Exogenous and endogenous glycolipid antigens activate NKT cells during microbial infections. Nature. (2005) 434:525–9. 10.1038/nature03408 15791258

[27] Kita H Naidenko OV Kronenberg M Ansari AA Rogers P He XS. Quantitation and phenotypic analysis of natural killer T cells in primary biliary cirrhosis using a human CD1d tetramer. Gastroenterology. (2002) 123:1031–43. 10.1053/gast.2002.36020 12360465

[28] Harada K Isse K Tsuneyama K Ohta H Nakanuma Y. Accumulating CD57 + CD3 + natural killer T cells are related to intrahepatic bile duct lesions in primary biliary cirrhosis. Liver Int. (2003) 23:94–100. 10.1034/j.1600-0676.2003.00807.x 12654131

[29] Tsuneyama K Yasoshima M Harada K Hiramatsu K Gershwin ME Nakanuma Y. Increased CD1d expression on small bile duct epithelium and epithelioid granuloma in livers in primary biliary cirrhosis. Hepatology. (1998) 28:620–3. 10.1002/hep.510280303 9731549

[30] Wynn TA. Cellular and molecular mechanisms of fibrosis. J Pathol. (2008) 214:199–210. 10.1002/path.2277 18161745 PMC2693329

[31] Delovitch TL Singh B. The nonobese diabetic mouse as a model of autoimmune diabetes: immune dysregulation gets the NOD. Immunity. (1997) 7:727–38. 10.1016/S1074-7613(00)80392-1 9430219

[32] Zhang BB Yan C Fang F Du Y Ma R Li XY. Increased hepatic Th2 and Treg subsets are associated with biliary fibrosis in different strains of mice caused by Clonorchis sinensis. PLoS ONE. (2017) 12:e0171005. 10.1371/journal.pone.0171005 28151995 PMC5289492

[33] Shi T Zhang T Zhang L Yang Y Zhang H Zhang F. The distribution and the fibrotic role of elevated inflammatory Th17 cells in patients with primary biliary cirrhosis. Medicine. (2015) 94:e1888. 10.1097/MD.0000000000001888 26554784 PMC4915885

[34] Qian C Jiang T Zhang W Ren C Wang Q Qin Q. Increased IL-23 and IL-17 expression by peripheral blood cells of patients with primary biliary cirrhosis. Cytokine. (2013) 64:172–80. 10.1016/j.cyto.2013.07.005 23910013

[35] Yang CY Ma X Tsuneyama K Huang S Takahashi T Chalasani NP. IL-12/Th1 and IL-23/Th17 biliary microenvironment in primary biliary cirrhosis: implications for therapy. Hepatology. (2014) 59:1944–53. 10.1002/hep.26979 24375552 PMC3999171

[36] Chen SS Sun LW Brickner H Sun PQ. Downregulating galectin-3 inhibits proinflammatory cytokine production by human monocyte-derived dendritic cells via RNA interference. Cell Immunol. (2015) 294:44–53. 10.1016/j.cellimm.2015.01.017 25684095 PMC4704704

[37] Volarevic V Milovanovic M Ljujic B Pejnovic N Arsenijevic N Nilsson U. Galectin-3 deficiency prevents concanavalin A-induced hepatitis in mice. Hepatology. (2012) 55:1954–64. 10.1002/hep.25542 22213244

[38] Jeftic I Jovicic N Pantic J Arsenijevic N Lukic ML Pejnovic N. Galectin-3 ablation enhances liver steatosis, but attenuates inflammation and IL-33-dependent fibrosis in obesogenic mouse model of nonalcoholic steatohepatitis. Mol Med. (2015) 21:453–65. 10.2119/molmed.2014.00178 26018806 PMC4559528

[39] Volarevic V Markovic BS Bojic S Stojanovic M Nilsson U Leffler H. Gal-3 regulates the capacity of dendritic cells to promote NKT-cell-induced liver injury. Eur J Immunol. (2015) 45:531–43. 10.1002/eji.201444849 25359399

[40] Collins PM Bum-Erdene K Yu X Blanchard H. Galectin-3 interactions with glycosphingolipids. J Mol Biol. (2014) 426:1439–51. 10.1016/j.jmb.2013.12.004 24326249

[41] Wree A Eguchi A McGeough MD Pena CA Johnson CD Canbay A. NLRP3 inflammasome activation results in hepatocyte pyroptosis, liver inflammation, and fibrosis in mice. Hepatology. (2014) 59:898–910. 10.1002/hep.26592 23813842 PMC4008151

[42] Szabo G Petrasek J. Inflammasome activation and function in liver disease. Nat Rev Gastroenterol Hepatol. (2015) 12:387–400. 10.1038/nrgastro.2015.94 26055245

[43] Seya T Akazawa T Tsujita T Matsumoto M. Role of Toll-like receptors in adjuvant-augmented immune therapies. Evid Based Complement Alternat Med. (2006) 3:31–8. 10.1093/ecam/nek010 16550221 PMC1375233

[44] Li Y Komai-Koma M Gilchrist DS Hsu DK Liu FT Springall T. Galectin-3 is a negative regulator of lipopolysaccharide-mediated inflammation. J Immunol. (2008) 181:2781–9. 10.4049/jimmunol.181.4.2781 18684969

