# Supplementary material for: Gal-3 Deficiency Suppresses Novosphyngobium aromaticivorans Inflammasome Activation and IL-17 Driven Autoimmune Cholangitis in Mice
Source: Front Immunol. 2019 Jun 7;10:1309. doi: 10.3389/fimmu.2019.01309 (PMC6568238; doi:10.3389/fimmu.2019.01309)

Supplemental figure 1. Gating strategy for identifying IL-17 and IFN-γ positive cells in CD4+ and CD8+ populations.

Mononuclear cells were gated. Then CD4+ and CD8+ cells were gated and percentages of IL-17 and IFN-γ positive cells in CD4+ and CD8+ populations were detected. Representative plots with percentages of IL-17 and IFN-γ positive cells in CD4+ and CD8+ populations in *N. aro* infected C57BL/6 WT and Lgals3-/- mice are presented.

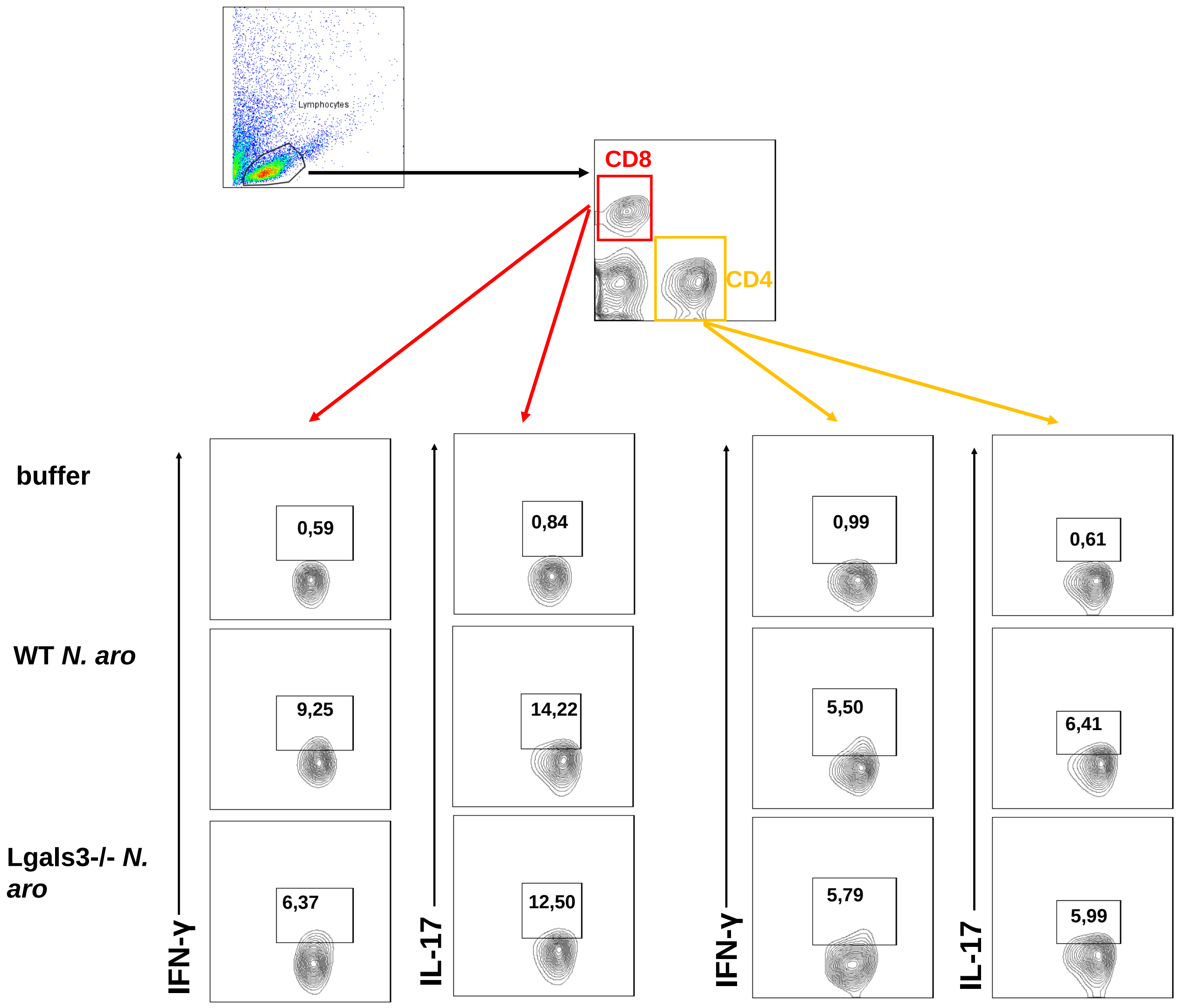

Supplemental figure 2. Gating strategy related to Figure 4.

Gating strategy for CD11c+ cells, double positive cells CD11c+CD11b+, CD11c+CD1d+, and CD11c+CD86+ cells and representative plots showing percenteges of these cells in uninfected and *N. aro* infected C57BL/6 WT and Lgals3-/- mice (A). Gating strategy for triple positive CD11c+CD11b+CD86+ and CD11c+CD1d+CD86+ cells with representative plots for *N. aro* infected C57BL/6 WT and Lgals3-/- mice (B). The same gating strategy was used for detection of cytokine (TNF-α, IL-12, and IL-6) expressing CD11c+CD11b+ and CD11c+CD1d+ cells.

A

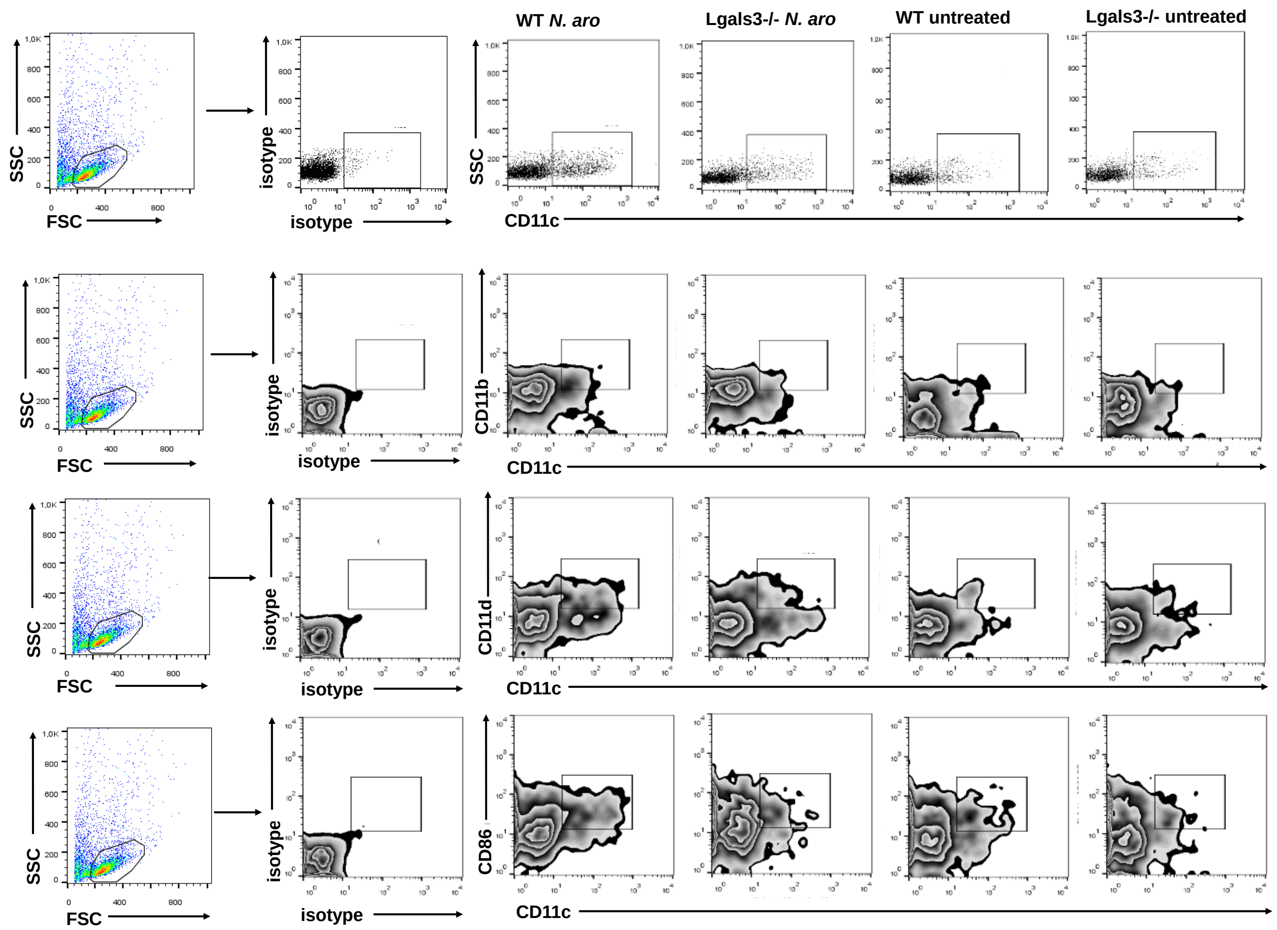

B

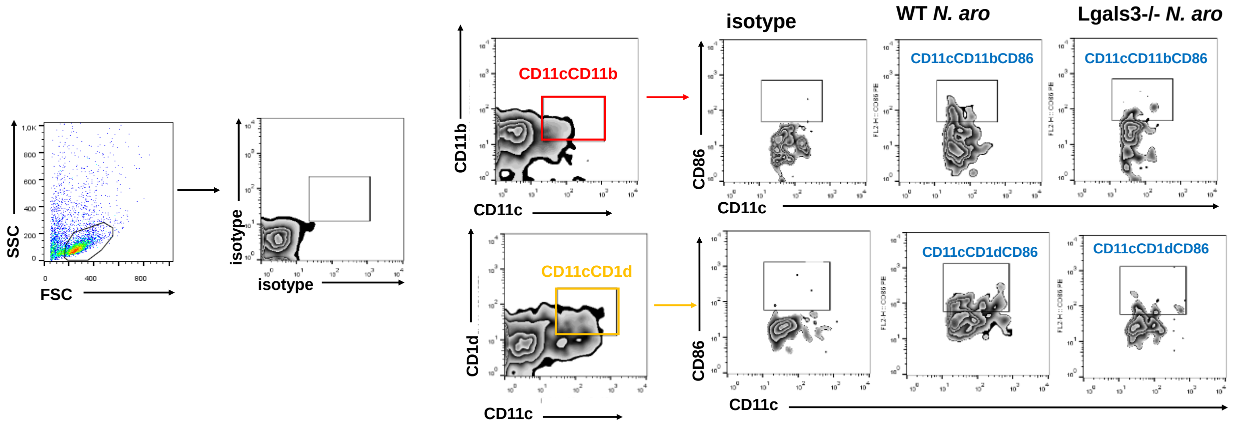

Supplemental figure 3. No primary antibody control related to Figure 8A and 8B
Immunohistochemical staining of liver sections incubated with only the antibody diluent without adding the primary antibody visualized by rabbit specific HRP/AEC detection IHC Kit (Abcam, Cambridge, UK).

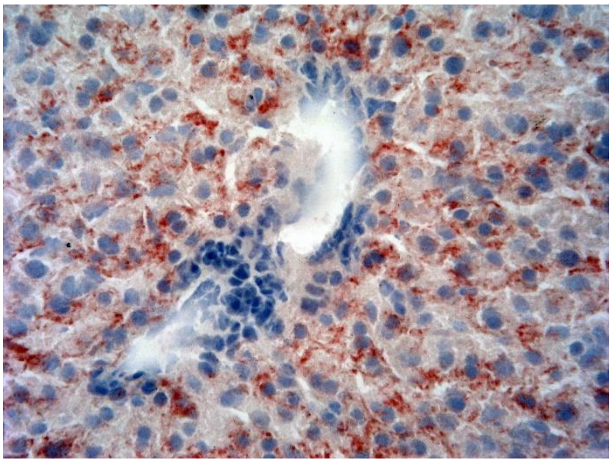

Supplemental figure 4. Gating strategy related to Figure 8C.
Gating strategy for NLRP3+, IL-1β, and IL-18 positive cells in population of F4/80 macrophages with representative plots for *N. aro* infected C57BL/6 WT and Lgals3-/- mice and Gal-3 INH treated mice.

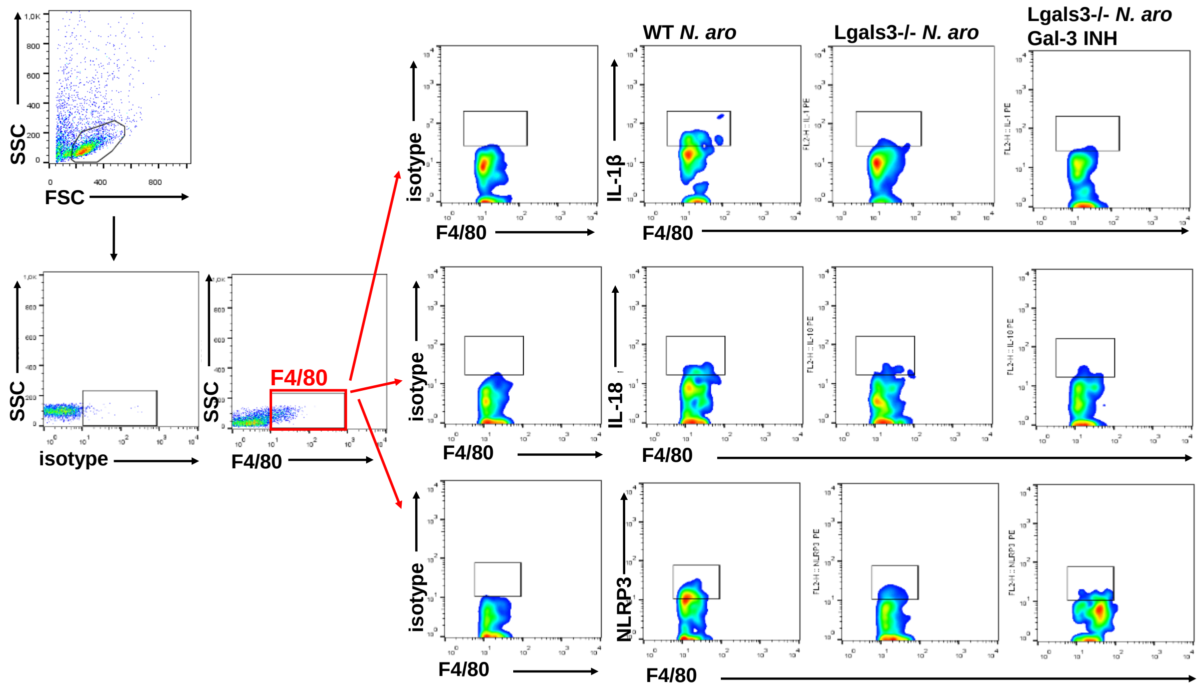

Supplement: Supplementary file 1 [file Data_Sheet_1.PDF]
